# Supplementary material for: Pneumococcal colonization prevalence and density among Thai children with severe pneumonia and community controls
Source: PLoS One. 2020 Apr 29;15(4):e0232151. doi: 10.1371/journal.pone.0232151 (PMC7190126; doi:10.1371/journal.pone.0232151)
Supplement: S1 Table — RTI, Respiratory Tract Illness; IQR, Inter quartile range; PCR, Polymerase chain reaction; NP, Nasopharyngeal; CRP, C-reactive protein; RSV, Respiratory syncytial virus; NP PCR, Nasopharyngeal Polymerase Chain Reaction; Flu A/B, Influenza A/B. a. Comparison of colonization prevalence: p = 0.01 in RTI controls vs Non-RTI controls. Comparison of median density: p < .0001 in RTI controls vs Non-RTI controls; p < .0001 in All cases vs RTI controls; p < 0.01 in All cases vs Non-RTI controls. b. Presence of antibiotics by serum. P-values calculated excluding those with parental report only. P-values obtained from logistic regression adjusted for age (pneumococcal colonization prevalence) and Kruskal-Wallis (pneumococcal density). Bolded p-values < 0.05. (PDF) [file pone.0232151.s001.pdf]

S1 Table. Pneumococcal nasopharyngeal PCR positivity and density among controls by acute respiratory illness symptoms.

Sub-study groups	RTI controls N=250					Non-RTI controls N=400				
Characteristics	N	*S. pneumoniae* colonization prevalence		*S. pneumoniae* Colonization density		N	*S. pneumoniae* colonization prevalence		*S. pneumoniae* Colonization density	
		n (%)	p-value[a]	Median (IQR) (10^3 copies/ml)	p-value[b]		n (%)	p-value[a]	Median (IQR) (10^3 copies/ml)	p-value[b]
**Overall[a]**	250	172 (68.8)		505.8 (93.5,1669.0)		400	234 (58.5)		109.5 (11.3,571.2)	
**Provincial site**										
**Nakhon Phanom**	160	113 (70.6)	0.48	695.1 (171.0,2163.1)	**0.017**	269	161 (59.9)	0.53	176.2 (19.9,862.9)	**0.0002**
**Sa Kaeo**	90	59 (65.6)		304.6 (70.0,1109.4)		131	73 (55.7)		27.9 (5.7,141.3)	
**Age**										
**1-5 months**	25	13 (52.0)	**0.01**	1,298.0 (566.1,3587.1)	0.13	65	27 (41.5)	**0.0001**	223.0 (19.9,1038.4)	0.84
**6-11 months**	61	41 (67.2)		628.2 (240.8,1569.6)		89	47 (52.8)		117.9 (16.3,571.2)	
**12-23 months**	81	50 (61.7)		550.3 (90.0,1526.7)		131	74 (56.5)		92.1 (10.3,822.6)	
**24-59 months**	83	68 (81.9)		318.0 (71.0,1629.6)		115	86 (74.8)		111.8 (9.4,415.5)	
**Gender**										
**Male**	126	85 (67.5)	0.62	485.3 (109.9,1526.7)	0.88	204	124 (60.8)	0.38	125.7 (17.1,551.1)	0.56
**Female**	124	87 (70.2)		526.2 (90.0,1943.7)		196	110 (56.1)		82.3 (7.8,623.4)	
**Prior antibiotic use**										
**Any documented antibiotic pretreatment prior to specimen collection[b]**	5	1 (20.0)	0.04	0.7 (--)	0.10	0	0 (0)	--	--	--
**Parental report only**	38	28 (73.7)		349.5 (33.5, 1501.7)		13	7 (58.9)		186.4 (16.3, 862.9)	
**No evidence of antibiotic use**	207	143 (69.1)		566.1 (112.8, 1754.3)		387	227 (58.7)		108.3 (10.6, 571.2)	

Sub-study groups	RTI controls N=250					Non-RTI controls N=400				
Characteristics	N	*S. pneumoniae* colonization prevalence		*S. pneumoniae* Colonization density		N	*S. pneumoniae* colonization prevalence		*S. pneumoniae* Colonization density	
**NP culture positive for pneumococcus**										
**Yes**	155	150 (96.8)	**<.0001**	671.8 (183.5,1871.7)	**<.0001**	185	179 (96.8)	**<.0001**	154.4 (24.9,822.6)	**<.0001**
**No**	95	22 (23.2)		6.6 (0.9,238.1)		214	54 (25.2)		6.5 (0.7,168.7)	
**CRP≥ 40 mg/L**										
**Yes**	0	0 (0)	--	--	--	0	0 (0)	--	--	--
**No**	45	36 (80.0)		1533.8 (267.3,3494.4)		43	25 (58.1)		168.7 (33.3,970. 6)	
**RSV NPPCR positive**										
**Yes**	14	12 (85.7)	0.20	582.5 (191. 9,2650.5)	0.80	5	4 (80.0)	0.31	19.7 (3.7,327.9)	0.28
**No**	236	160 (67.8)		505.8 (90.4,1668.9)		395	230 (58.2)		113.0 (12.0,571.2)	
**Flu A/B NPPCR positive**										
**Yes**	0	0 (0)	--	--	--	0	0 (0)	--	--	--
**No**	248	172 (69.4)		505.8 (93.5,1668.9)		400	234 (58.5)		109.5 (11.3,571.2)	
**Any virus NPPCR positive**										
**Yes**	214	147 (68.7)	0.77	566.1 (90.0,1718.3)	0.88	323	195 (60.4)	0.07	108.3 (10.3,642.6)	0.89
**No**	36	25 (69.4)		314.3 (112.8,954.9)		77	39 (50.6)		115.9 (24.4,303.1)	
**Whole blood *lyt* A PCR positive**										
**Yes**	4	3 (75.0)	0.82	3618. 9 (1754.3,4107.5)	**0.04**	1	1 (100)	--	3,017.5 (--)	0.12
**No**	236	165 (69.9)		481.9 (90.9,1540.8)		373	222 (59.5)		107.0 (10.3,553.2)	
**Whole blood *lytA* PCR density ≥ 2.2 log10 copies/ml**										

**Sub-study groups**	**RTI controls N=250**					**Non-RTI controls N=400**				
**Characteristics**	**N**	***S. pneumoniae* colonization prevalence**		***S. pneumoniae* Colonization density**		**N**	***S. pneumoniae* colonization prevalence**		***S. pneumoniae* Colonization density**	
**Yes**	2	2 (100)	--	2686.6 (1754.3,3618.9)	0.12	1	1 (100)	--	3,017.5 (--)	0.12
**No**	238	166 (69.7)		483.6 (90.9,1569.6)		373	222 (59.5)		107.0 (10.3,553.2)	
**Observed cough**										
**Yes**	110	75 (68.2)	0.94	481.9 (159.3,1569.6)	0.74	0	0 (0)	--	--	--
**No**	140	97 (69.3)		526.2 (77.4,1718.3)		400	234 (58.5)		109.5 (11.3,571.2)	

RTI, Respiratory Tract Illness; IQR, Inter quartile range; PCR, Polymerase chain reaction; NP, Nasopharyngeal; CRP, C-reactive protein; RSV, Respiratory syncytial virus; NP PCR, Nasopharyngeal Polymerase Chain Reaction; Flu A/B, Influenza A/B.

a. Comparison of colonization prevalence: $p = 0.01$ in RTI controls vs Non-RTI controls. Comparison of median density: $p < .0001$ in RTI controls vs Non-RTI controls; $p < .0001$ in All cases vs RTI controls; $p < 0.01$ in All cases vs Non-RTI controls.

b. Presence of antibiotics by serum. P-values calculated excluding those with parental report only.

P-values obtained from logistic regression adjusted for age (pneumococcal colonization prevalence) and Kruskal-Wallis (pneumococcal density). Bolded p-values < 0.05.
