# Supplementary material for: Pneumococcal colonization prevalence and density among Thai children with severe pneumonia and community controls
Source: PLoS One. 2020 Apr 29;15(4):e0232151. doi: 10.1371/journal.pone.0232151 (PMC7190126; doi:10.1371/journal.pone.0232151)
Supplement: S1 Fig — Bars represent the percent of cases or controls with the serotyped detected, restricted to children with NP culture serotyping data. Error bars are 95% Wald confidence intervals. PCV-10 serotypes: 1, 4, 5, 6B, 7F, 9V, 14, 18C, 19F, and 23F; PCV-13 serotypes: PCV-10 serotypes plus serotypes 3, 6A, and 19A. (PDF) [file pone.0232151.s002.pdf]

S1 Fig. Pneumococcal colonization serotype distribution among pneumonia cases and community controls in rural Thailand: Nakhon Phanom.

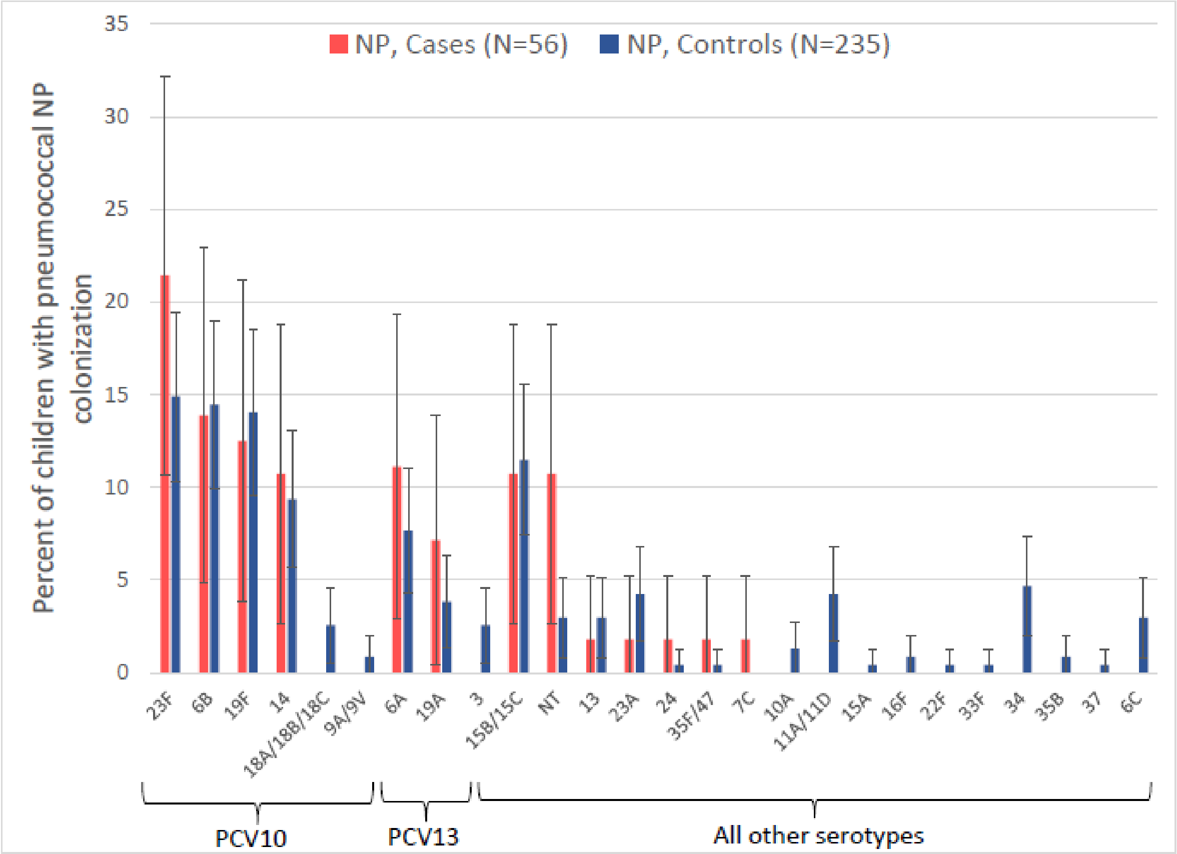

Bars represent the percent of cases or controls with the serotyped detected, restricted to children with NP culture serotyping data. Error bars are 95% Wald confidence intervals. PCV-10 serotypes: 1, 4, 5, 6B, 7F, 9V, 14, 18C, 19F, and 23F; PCV-13 serotypes: PCV-10 serotypes plus serotypes 3, 6A, and 19A.
