# Supplementary material for: Pneumococcal colonization prevalence and density among Thai children with severe pneumonia and community controls
Source: PLoS One. 2020 Apr 29;15(4):e0232151. doi: 10.1371/journal.pone.0232151 (PMC7190126; doi:10.1371/journal.pone.0232151)
Supplement: S1 Data — (PDF) [file pone.0232151.s004.pdf]

PERCH AGREEMENT FOR DATA, DISSEMINATION & SPECIMENS

PNEUMONIA ETIOLOGY RESEARCH FOR CHILD HEALTH

THIS AGREEMENT is entered into upon signing, by and among each of the following (each a "Party," and collectively the "Parties").

INSTITUTION OR CENTER	LOCATION	ROLE IN PERCH
Johns Hopkins University, on behalf of its Bloomberg School of Public Health / International Vaccine Access Center (IVAC)	Baltimore, MD, USA	Lead Institution and Project Coordinating Center
The Chancellor, Masters and Scholars of the University of Oxford (University of Oxford)	Oxford, United Kingdom	Field site for Kenya
Medical Research Council<br>Representing its Medical Research Council Unit, The Gambia	Fajara, The Gambia	Field site for The Gambia
University of Maryland Baltimore,<br>School of Medicine's Center for Vaccine Development (UMB)	Baltimore, MD, United States	Field site for Mali
Trustees of Boston University<br>(Boston University)	Boston, MA, United States	Field site for Zambia
Wits Health Consortium (Pty) Ltd (University of Witwatersrand)	Johannesburg, South Africa	Field site for South Africa
Thailand Ministry of Public Health - U.S. Centers for Disease Control and Prevention (CDC) Collaboration	Nonthaburi, Thailand	Field site for Thailand
The International Center for Diarrhoeal Diseases Research, Bangladesh (ICDDR,B)	Dhaka, Bangladesh	Field site for Bangladesh
University of Otago, a body corporate established under the University of Otago Ordinance 1869, the University of Otago Amendment Act 1961 and the Education Act 1989, of Dunedin, New Zealand (University of Otago)	Christchurch, New Zealand	Laboratory support for Project
Canterbury Health Laboratories (CHL)	Christchurch, New Zealand	Laboratory support for Project

BACKGROUND

WHEREAS, each Party is a member of the Pneumonia Etiology Research for Child Health Study Consortium (the **"PERCH Consortium"**), which is a network of research institutes, centers and academic units created to collaborate in the implementation of the scientific research plan of the PERCH study, namely the creation of knowledge on the etiology of and risk factors associated with severe and very severe pneumonia among infants and young children in developing countries, in order to provide a basis for the sound implementation of existing treatments and interventions, and investment in the highest priority needed new interventions (the **"PERCH Project"**).

WHEREAS, the PERCH Project is funded by a grant from The Bill & Melinda Gates Foundation (the **"Foundation"**) to The Johns Hopkins University, on behalf of its Bloomberg School of Public Health and the International Vaccine Access Center pursuant to Grant Number OPP48968 (entitled "Pneumonia Etiology Research for Child Health (PERCH) Project").

WHEREAS, the PERCH Consortium is not a separate legal entity, and its members include public and private entities.

WHEREAS, it is expected that the Parties will work in collaboration as PERCH Consortium Members, united by a robust system for the exchange of PERCH Data and Materials (as defined in Section 1 below).

WHEREAS, each of the Parties acknowledges that the primary goals of the PERCH Project are as follows, and achievement of these goals shall be conducted in a manner that is consistent with and in furtherance of the Global Access Objectives (as defined in Section 1 below):

(i) To determine the association between pneumonia and infection with known and putative viral, bacterial, mycobacterial, and fungal pathogens;

(ii) To estimate the fraction of pneumonia attributable to pathogens for which vaccines are currently under development (including *S. pneumoniae* common protein vaccines, respiratory syncytial virus (RSV), parainfluenza virus (PIV), influenza and *Staphylococcus aureus*) as well as other known, but poorly quantified causes of pneumonia in children including, but not limited to, non-typeable *H. influenzae*, nontyphoidal *Salmonella*, human metapneumovirus, *M. tuberculosis*, *Pneumocystis jiroveci*, and potentially fastidious bacteria;

(iii) To assess putative risk factors for infection and/or disease particularly due to novel or under-recognized pneumonia pathogens;

(iv) To determine the association between disease severity and etiology;

(v) To develop a set of specimens for novel pathogen discovery among episodes with no known etiology (negative with comprehensive testing);

(vi) To determine patterns of antimicrobial resistance among invasive isolates including, but not limited to, *M. tuberculosis*, *S. aureus*, and *S. pneumoniae*;

(vii) To develop a set of isolates of key pathogens associated with pneumonia including, but not limited to, influenza, *S. pneumoniae*, nontyphoidal *Salmonella*, and *S. aureus* for molecular epidemiologic analyses;

(viii) To develop a robust clinical severity index based on analyses of PERCH putative criteria and outcomes;

(ix) To provide a robust platform for ancillary studies of pneumonia epidemiology including, but not limited to, the utility of digital auscultation, chest radiograph, and viral quantification.

NOW, THEREFORE, each of the Parties agrees to the following terms and conditions:

§1. **<u>Definitions</u>.** In this Agreement (including without limitation the Annexes), the following terms have the meanings set forth in this Article:

"BMGF" or "The Foundation": The Bill & Melinda Gates Foundation.

"Commercial Purposes" means the use or transfer of Materials, PERCH Data, or Confidential Information by, to, on behalf of, or for research sponsored by a for-profit company.

"Confidential Information" has the meaning as provided in the Master PERCH Confidential Disclosure Agreement, attached hereto as **Exhibit B**.

"Coordinating Principal Investigator (PI)": Dr. Katherine O'Brien of the International Vaccine Access Center, Johns Hopkins Bloomberg School of Public Health.

"Co-Principal Investigator (Co-PI)": Dr. Maria Deloria-Knoll of the International Vaccine Access Center, Johns Hopkins Bloomberg School of Public Health and Dr. Anthony Scott of the London School of Hygiene and Tropical Medicine.

"**IVAC**": International Vaccine Access Center of The Johns Hopkins Bloomberg School of Public Health.

"Data Coordination Center" means The Emmes Corporation, located in Rockville, Maryland, USA.

"Data Freeze Datasets" means a dataset obtained at a specified timepoint that contains data ready and amenable to analyses. The "frozen" dataset will be made available to investigators and groups to carry out analyses. The main data freezes will occur prior to the publication of the foundational analyses and prior to the publication of the primary analyses. Subsequent, sequential data freezes may occur to incorporate additional lab testing or address data cleaning issues identified during analyses. See **Annex A1** for additional details.

"**External Investigator"** means an investigator who is not an investigator within the PERCH Consortium.

"Global Access Objectives" means (i) the prompt dissemination of new scientific information within the PERCH Consortium and with the broader scientific community and (ii) facilitating the accessibility of data and Materials from the study repository following the data freeze for the primary publication, based on criteria specified in **Annex A1 and Annex A2**.

"**Good Clinical Data Management Practices"** means the Good Clinical Data Management Practices promulgated by the Society for Clinical Data Management, as they may be amended from time to time. These are available at http://www.scdm.org/sitecore/content/be-bruga/scdm/Publications/gcdmp.aspx

"**IRB"** means Institutional Review Board or comparable authority (e.g., Ethics Review Committee), in the Party's home jurisdiction.

“Materials” means specimens, including whole blood, sera, upper respiratory swabs, induced sputum, endotracheal tube aspirates, lung aspirate, pleural fluid, post mortem lung tissue, urine, bacterial isolates, nucleic acids, and other biological materials accumulated in the performance of the PERCH Project.

“PADDS” is the acronym for the PERCH Agreement for Data, Dissemination & Specimens.

“PCAP” means **PERCH Consortium Analysis Period**, which is the period of 36 months following the data freeze for the primary publication. During this time, the PERCH Consortium will focus on accomplishing analyses to address the primary and secondary analyses of the PERCH Project. Data access to investigators may be granted following review of a proposal by the PERCH Data Access Committee and site-level review committees, as required by the site, and through engagement and meaningful collaboration with PERCH Consortium members, as set forth in **Annex A1**.

“PEAP” means **PERCH Extended Analysis Period**, which is the period of 60 months following the PCAP, as set forth in **Annex A1**.

“PERCH” means the “Pneumonia Etiology Research for Child Health.”

“**PERCH Biorepository Specimen and Access Committee”** means a committee comprised of the PERCH Executive Committee (EC) and a representative of the BMGF who will review all specimen requests, as set forth in **Annex A2**.

“**PERCH Consortium Members**” means the Parties, including all investigators affiliated with the Parties who are or were engaged with the PERCH Project. Membership in the PERCH Consortium may change and is at the discretion of the Executive Committee.

“PERCH Consortium Specimen Repository” has the meaning set forth in **Annex A2**.

“PERCH Core” means the investigators from the International Vaccine Access Center of The Johns Hopkins Bloomberg School of Public Health and from the University of Otago, who coordinate the PERCH Project and serve as a link among all PERCH Consortium Members.

“**PERCH Data**” means all data used or generated in the performance of the PERCH Project.

“PERCH Data Access Committee” means a committee comprised of the PERCH Executive Committee (EC) and a representative of the BMGF who will review all data access requests, as set forth in **Annex A1**.

“**PERCH Datasets”** include the Raw Datasets and Analytic Datasets generated in the performance of the PERCH Project. See **Appendix I** for the complete list of datasets.

“**PERCH Executive Committee (EC)**” shall consist of the following members, including three (3) Core team members and the seven (7) site PIs or representative:

**PERCH Executive Committee**	
**Members**	**Role & Responsibility**
Dr. Katherine O’Brien	PI
Dr. Daniel Feikin	Core Team Secretariat Member – Clinical/Epidemiology
Dr. David Murdoch	Core Team Secretariat Member – Laboratory
Dr. Anthony Scott	Field site PI: Kenya
Dr. Syed Zaman	Field site PI: The Gambia
Dr. Karen Kotloff	Field site PI: Mali

Dr. Donald Thea	Field site PI: Zambia
Dr. Shabir Madhi	Field site PI: South Africa
Dr. Henry (Kip) Baggett	Field site PI: Thailand
Dr. Abdullah Brooks	Field site investigator: Bangladesh

“PERCH Expert Group” refers to the external group of experts who provide independent expert advice in relation to the PERCH project. The group should be acknowledged in manuscripts developed using PERCH Data or Materials, set forth in **Annex A3**.

“PERCH Invention” means a creation or invention which is made directly in connection with performance of the PERCH Project, and which is or may be patentable or otherwise protected or protectable under law as intellectual property.

“PERCH Investigator” means an investigator involved in the PERCH Consortium.

“PERCH Sites” means the field sites where children were enrolled in the PERCH Project and where data were collected. These include Kenya (Kilifi), The Gambia (Basse), Mali (Bamako), Zambia (Lusaka), South Africa (Soweto), Thailand (Nakhon Phanom and Sa Kaeo) and Bangladesh (Dhaka and Matlab).

“PERCH Study Group” means the authorship group to be acknowledged in all manuscripts developed using PERCH Data or Materials, either as an author or in the Acknowledgements section, as set forth in **Annex A3**. The group consists of members from the PERCH Consortium.

“**PI**” means Principal Investigator.

“**Protocols**” means the most current IRB-approved version and all previous approved versions of the clinical protocol entitled “The Pneumonia Etiology Research for Child Health Project (PERCH).”

“Reference Laboratory” means the following, which is party to this agreement:

Canterbury Health Laboratories
Cnr Tuam & Hagley Ave
Opposite Christchurch Hospital
Christchurch
New Zealand
Phone: + (64) 3-364-0300
Fax: + (64) 3-364-0750

“Site Principal Investigator” or **“Site PI”** means the PI for a field site, which may change from time to time. At the time of signature, the Site PIs are as follows:

Principal Investigator	Role	Contact Information
Dr. Anthony Scott	Field site PI: Kenya	ascott@ikilifi.org
Dr. Syed Zaman	Field site PI: The Gambia	azaman@mrc.gm
Dr. Karen Kotloff	Field site PI: Mali	Kkotloff@medicine.umaryland.edu
Dr. Donald Thea	Field site PI: Zambia	dthea@bu.edu
Dr. Shabir Madhi	Field site PI: South Africa	ShabirM@nicd.ac.za
Dr. Henry (Kip) Baggett	Field site PI: Thailand	hfb8@cdc.gov
Dr. Doli Goswami	Field site PI: Bangladesh	drdolly@icddrb.org

2. **<u>PERCH Agreement for Data, Dissemination & Specimens.</u>** During the course of performing the PERCH Project, each Party agrees to comply with and to perform in accordance with each of the following:

a. PERCH Consortium Data Sharing Agreement, regarding the sharing of PERCH Data (**"Annex A1"**);

b. PERCH Consortium Material Sharing Agreement, regarding the specimen repository and sharing of Materials (**"Annex A2"**); and

c. PERCH Consortium Publication Agreement (**"Annex A3"**).

Thus, the Agreement contains three parts that cover: i) PERCH Data access and sharing; ii) Materials access and sharing; and iii) Guidelines for publications, including eligibility for writing manuscripts and authorship.

3. **<u>Protocols</u>**. Each Party agrees to perform its duties and obligations in connection with the PERCH Project in accordance with the IRB Approved Protocols.

4. **<u>Good Clinical Data Management Practices</u>**.

(a) During the course of performing the PERCH Project, each Party agrees to comply with Good Clinical Data Management Practices.

(b) Moreover, when PERCH Data become available to the broad scientific community during and following the PERCH Consortium Analysis Period (see **Annex A1**), External Investigators and PERCH Consortium Members receiving data from sites they are not affiliated with will be required to agree to handle the PERCH Datasets in accordance with Good Clinical Data Management Practices.

5. **<u>Material Transfer Agreement</u>**. Each Party agrees that Materials may only be transferred from a PERCH Consortium Member to an External Investigator or between PERCH Consortium Members in accordance with the Material Transfer Agreement, in the form attached as **Exhibit A** (the **"Material Transfer Agreement "**).

6. **<u>Confidential Disclosure Agreement</u>**. Each Party agrees that Confidential Information may only be exchanged between a PERCH Consortium Member and an External Investigator or between PERCH Consortium Members in accordance with the terms of the Confidential Disclosure Agreement, in the form attached as **Exhibit B** (the "**Confidential Disclosure Agreement**"). The purpose of the Confidential Disclosure Agreement is to require the investigators to use PERCH Data only for the research purposes specified in the investigator's request and to keep confidential any PERCH Data or other confidential information received from a PERCH Consortium Member outside of the scope of the investigator's specified research.

7. **<u>Ownership.</u>** Each Party agrees that all PERCH Data and Materials produced by or at a site are the property of the PERCH Consortium Member which produced the relevant PERCH Data or Materials. By signing this Agreement, each Site PI acknowledges that ownership by the PERCH Consortium Member which employs him/her, and acknowledges that he/she has no personal ownership of the relevant PERCH Data or Materials.

8. **<u>Public Announcements.</u>** Each Party (other than The Johns Hopkins University) has been selected to participate in the PERCH Project at the discretion of The Johns Hopkins University. No Party (other than The Johns Hopkins University) may make any statement or otherwise imply to donors, investors, media or the general public that it is a direct grantee of the

Bill & Melinda Gates Foundation. No party shall use directly or by implication the names of the other parties, nor any of the other parties affiliates or contractors, nor any abbreviations thereof, or of any staff member, faculty member, student, or employee of the other parties in connection with any products, publicity, promotion, financing, advertising, or other public disclosure without the prior written permission of the other party.

9. **Annexes.** All Annexes are incorporated into and made a part of this Agreement.

10. **Dispute Resolution.** All disputes under this Agreement shall be resolved and conducted, regardless of the means or authority, in the English language. Any dispute or controversy arising in connection with this Agreement shall first be referred to the parties' respective officers that signed this document, on behalf of the Parties, or their successors, for attempted resolution in good faith negotiations within sixty (60) days of notice of such dispute. If such officers are not able to resolve the dispute within the sixty (60) day period, or any agreed upon extensions, the Parties shall be free to resolve the dispute through any dispute resolution mechanism they may individually or collectively choose. Each party agrees that, prior to resorting to litigation to resolve any dispute, it will confer with the other party to determine whether other procedures that are less expensive or less time consuming can be adopted to resolve the dispute.

11. **Counterparts.** This Agreement may be signed in counterparts, each of which will be considered an original, and all of which collectively will be deemed the same document. Copies of this Agreement will be equally binding as originals and faxed or scanned and emailed counterpart signatures will be sufficient to evidence execution.

12. **Electronic Signatures.** This Agreement may be signed and delivered, or a signature may be transmitted or communicated, by means of facsimile or other electronic transmission (such as a Portable Document Format [PDF] copy of an original signature). In that event, this Agreement will be treated in for all purposes as an original agreement, and will be considered to have the same binding legal effect as if it were the original signed version delivered in person. No Party will raise the means of electronic transmission to deliver a signature or the fact that any signature or agreement was transmitted or communicated through the use of a means of electronic transmission as a defense to the formation or enforceability of a contract, and each Party agrees to forever waive any such defense.

13. **Amendments.** This Agreement may not be amended unless the amendment is in writing and signed by a duly authorized representative of each Party.

14. **Termination.** This Agreement shall remain in effect until March 31st 2024, which represents a period of eight (8) years following the data freeze on the primary publications. The duration of this Agreement may be extended by mutual agreement of the Parties.

15. **Order of Precedence.** To the extent that there is any conflict between provisions of this agreement and any subsequent subagreement issued by The Johns Hopkins University to a Party regarding the PERCH Project, the provisions of the subagreement shall take precedence.

[Signatures on following page]

SIGNATURE PAGES
to the
PERCH AGREEMENT FOR DATA, DISSEMINATION & SPECIMENS

IN WITNESS WHEREOF, the undersigned Parties execute the PERCH Data and Specimen Sharing Agreement.

JOHNS HOPKINS UNIVERSITY, BALTIMORE
ON BEHALF OF ITS BLOOMBERG SCHOOL OF PUBLIC HEALTH AND ITS INTERNATIONAL VACCINE ACCESS CENTER

By: ______________________________

Name: Jennifer Barron

Title: Executive Director

Date: 4/13/2016

Acknowledged by PERCH PI (Dr. Katherine O'Brien):

By: ______________________________

Date: 04/18/2016

THE CHANCELLOR, MASTERS AND SCHOLARS OF THE UNIVERSITY OF OXFORD

By: ______________________________

Name: ______________________________

Title: ______________________________

Date: ______________________________

Acknowledged by Site PI (Dr. Anthony Scott):

By: ______________________________

Date: ______________________________

MEDICAL RESEARCH COUNCIL REPRESENTING ITS MEDICAL RESEARCH COUNCIL UNIT, THE GAMBIA

By: ______________________________

Name: ______________________________

Title: ______________________________

Date: ______________________________

Acknowledged by Site PI (Dr. Syed Zaman):

By: ______________________________

Date: ______________________________

UNIVERSITY OF MARYLAND BALTIMORE, SCHOOL OF MEDICINE'S CENTER FOR VACCINE DEVELOPMENT (UMB)

By: ______________________________

Name: ______________________________

Title: ______________________________

Date: ______________________________

Acknowledged by Site PI (Dr. Karen Kotloff):

By: ______________________________

Date: ______________________________

Acknowledged by Site Representative (Dr. Samba Sow):

By: ______________________________

Date: ______________________________

SIGNATURE PAGES

to the

PERCH AGREEMENT FOR DATA, DISSEMINATION & SPECIMENS

IN WITNESS WHEREOF, the undersigned Parties execute the PERCH Data and Specimen Sharing Agreement.

**JOHNS HOPKINS UNIVERSITY, BALTIMORE**<br>*ON BEHALF OF ITS BLOOMBERG SCHOOL OF PUBLIC HEALTH AND ITS INTERNATIONAL VACCINE ACCESS CENTER*<br><br>By: ______________________<br>Name: ______________________<br>Title: ______________________<br>Date: ______________________<br><br>*Acknowledged by PERCH PI (Dr. Katherine O'Brien):*<br>By: ______________________<br>Date: ______________________	**THE CHANCELLOR, MASTERS AND SCHOLARS OF THE UNIVERSITY OF OXFORD**<br><br>By: [signature]<br>Name: Dr Richard Liwicki<br>Title: Deputy Director Research Services University of Oxford<br>Date: 14/iv/16<br><br>*Acknowledged by Site PI (Dr. Anthony Scott):*<br>By: Anthony Scott [signature]<br>Date: 14th April 2016
**MEDICAL RESEARCH COUNCIL REPRESENTING ITS MEDICAL RESEARCH COUNCIL UNIT, THE GAMBIA**<br><br>By: ______________________<br>Name: ______________________<br>Title: ______________________<br>Date: ______________________<br><br>*Acknowledged by Site PI (Dr. Syed Zaman):*<br>By: ______________________<br>Date: ______________________	**UNIVERSITY OF MARYLAND BALTIMORE, SCHOOL OF MEDICINE'S CENTER FOR VACCINE DEVELOPMENT (UMB)**<br><br>By: ______________________<br>Name: ______________________<br>Title: ______________________<br>Date: ______________________<br><br>*Acknowledged by Site PI (Dr. Karen Kotloff):*<br>By: ______________________<br>Date: ______________________<br><br>*Acknowledged by Site Representative (Dr. Samba Sow):*<br>By: ______________________<br>Date: ______________________

SIGNATURE PAGES

to the

PERCH AGREEMENT FOR DATA, DISSEMINATION & SPECIMENS

IN WITNESS WHEREOF, the undersigned Parties execute the PERCH Data and Specimen Sharing Agreement.

**JOHNS HOPKINS UNIVERSITY, BALTIMORE**<br>*ON BEHALF OF ITS BLOOMBERG SCHOOL OF PUBLIC HEALTH AND ITS INTERNATIONAL VACCINE ACCESS CENTER*<br><br>By: ______________<br>Name: ______________<br>Title: ______________<br>Date: ______________<br>*Acknowledged by PERCH PI (Dr. Katherine O'Brien):*<br>By: ______________<br>Date: ______________	**THE CHANCELLOR, MASTERS AND SCHOLARS OF THE UNIVERSITY OF OXFORD**<br><br>By: ______________<br>Name: ______________<br>Title: ______________<br>Date: ______________<br>*Acknowledged by Site PI (Dr. Anthony Scott):*<br>By: ______________<br>Date: ______________
**MEDICAL RESEARCH COUNCIL REPRESENTING ITS MEDICAL RESEARCH COUNCIL UNIT, THE GAMBIA**<br>By: ______________<br>Name: U. D'ALESSANDRO<br>Title: Unit Director<br>Date: 23/04/2016<br>*Acknowledged by Site PI (Dr. Syed Zaman):*<br>By: ______________<br>Date: 25/04/2016	**UNIVERSITY OF MARYLAND BALTIMORE, SCHOOL OF MEDICINE'S CENTER FOR VACCINE DEVELOPMENT (UMB)**<br>By: ______________<br>Name: ______________<br>Title: ______________<br>Date: ______________<br>*Acknowledged by Site PI (Dr. Karen Kotloff):*<br>By: ______________<br>Date: ______________<br>*Acknowledged by Site Representative (Dr. Samba Sow):*<br>By: ______________<br>Date: ______________

SIGNATURE PAGES
to the
PERCH AGREEMENT FOR DATA, DISSEMINATION & SPECIMENS

IN WITNESS WHEREOF, the undersigned Parties execute the PERCH Data and Specimen Sharing Agreement.

**JOHNS HOPKINS UNIVERSITY, BALTIMORE**<br>*ON BEHALF OF ITS BLOOMBERG SCHOOL OF PUBLIC HEALTH AND ITS INTERNATIONAL VACCINE ACCESS CENTER*<br><br>By: ______________________<br>Name: ______________________<br>Title: ______________________<br>Date: ______________________<br>*Acknowledged by PERCH PI (Dr. Katherine O'Brien):*<br>By: ______________________<br>Date: ______________________	**THE CHANCELLOR, MASTERS AND SCHOLARS OF THE UNIVERSITY OF OXFORD**<br><br>By: ______________________<br>Name: ______________________<br>Title: ______________________<br>Date: ______________________<br>*Acknowledged by Site PI (Dr. Anthony Scott):*<br>By: ______________________<br>Date: ______________________
**MEDICAL RESEARCH COUNCIL REPRESENTING ITS MEDICAL RESEARCH COUNCIL UNIT, THE GAMBIA**<br><br>By: ______________________<br>Name: ______________________<br>Title: ______________________<br>Date: ______________________<br>*Acknowledged by Site PI (Dr. Syed Zaman):*<br>By: ______________________<br>Date: ______________________	**UNIVERSITY OF MARYLAND BALTIMORE, SCHOOL OF MEDICINE'S CENTER FOR VACCINE DEVELOPMENT (UMB)**<br><br>By: Dennis J Paffrath<br>Name: Dennis J. Paffrath, MBA<br>Title: Assistant Vice President, SPA<br>Date: 4/14/16<br>*Acknowledged by Site PI (Dr. Karen Kotloff):*<br>By: Karen L Kotloff<br>Date: 04/12/2016<br>*Acknowledged by Site Representative (Dr. Samba Sow):*<br>By: Samba Ous Sow<br>Date: April 13. 2016

SIGNATURE PAGES
to the
PERCH AGREEMENT FOR DATA, DISSEMINATION & SPECIMENS

**TRUSTEES OF BOSTON UNIVERSITY**<br>By: [signature]<br>Name: William Segarra<br>Associate Director<br>Industry Contracts & Agreements<br>Title:<br>Date: 4/14/2016<br>*Acknowledged by Site PI (Dr. Donald Thea):*<br>By: [signature]<br>Date: 4/14/16	**THE INTERNATIONAL CENTER FOR DIARRHOEAL DISEASES RESEARCH, BANGLADESH (ICDDR,B)**<br>By:<br>Name:<br>Title:<br>Date:<br>*Acknowledged by Site PI (Dr. Doli Goswami):*<br>By:<br>Date:
**WITS HEALTH CONSORTIUM (PTY) LTD**<br>By:<br>Name:<br>Title:<br>Date:<br>*Acknowledged by Site PI (Dr. Shabir Madhi):*<br>By:<br>Date:	**THAILAND MINISTRY OF PUBLIC HEALTH – U.S. CENTERS FOR DISEASE CONTROL AND PREVENTION COLLABORATION**<br>By:<br>Name:<br>Title:<br>Date:<br>*Acknowledged by Site PI (Dr. Henry 'Kip' Baggett):*<br>By:<br>Date:

SIGNATURE PAGES
to the
PERCH AGREEMENT FOR DATA, DISSEMINATION & SPECIMENS

TRUSTEES OF BOSTON UNIVERSITY

By: ______________________

Name: ______________________

Title: ______________________

Date: ______________________

Acknowledged by Site PI (Dr. Donald Thea):

By: ______________________

Date: ______________________

THE INTERNATIONAL CENTER FOR DIARRHOEAL DISEASES RESEARCH, BANGLADESH (ICDDR,B)

By: [signature]

Name: DR. ABBAS BHUIYA

Title: Acting Executive Director

Date: 18 April 2016

Acknowledged by Site PI (Dr. Doli Goswami):

By: [signature]

Date: 18 April 2016

WITS HEALTH CONSORTIUM (PTY) LTD

By: ______________________

Name: ______________________

Title: ______________________

Date: ______________________

Acknowledged by Site PI (Dr. Shabir Madhi):

By: ______________________

Date: ______________________

THAILAND MINISTRY OF PUBLIC HEALTH –U.S. CENTERS FOR DISEASE CONTROL AND PREVENTION COLLABORATION

By: ______________________

Name: ______________________

Title: ______________________

Date: ______________________

Acknowledged by Site PI (Dr. Henry 'Kip' Baggett):

By: ______________________

Date: ______________________

SIGNATURE PAGES
to the
PERCH AGREEMENT FOR DATA, DISSEMINATION & SPECIMENS

**TRUSTEES OF BOSTON UNIVERSITY**<br><br>By: ______<br>Name: ______<br>Title: ______<br>Date: ______<br>*Acknowledged by Site PI (Dr. Donald Thea):*<br>By: ______<br>Date: ______	**THE INTERNATIONAL CENTER FOR DIARRHOEAL DISEASES RESEARCH, BANGLADESH (ICDDR,B)**<br><br>By: ______<br>Name: ______<br>Title: ______<br>Date: ______<br>*Acknowledged by Site PI (Dr. Doli Goswami):*<br>By: ______<br>Date: ______
**WITS HEALTH CONSORTIUM (PTY) LTD**<br><br>By: [signature]<br>Name: Alfred Farrell<br>Title: Chief Executive Officer Wits Health Consortium (Pty) Ltd<br>Date: 12 APR 2016.<br>*Acknowledged by Site PI (Dr. Shabir Madhi):*<br>By: [signature]<br>Date: 14 APR 16	**THAILAND MINISTRY OF PUBLIC HEALTH – U.S. CENTERS FOR DISEASE CONTROL AND PREVENTION COLLABORATION**<br><br>By: ______<br>Name: ______<br>Title: ______<br>Date: ______<br>*Acknowledged by Site PI (Dr. Henry 'Kip' Baggett):*<br>By: ______<br>Date: ______

SIGNATURE PAGES

to the

PERCH AGREEMENT FOR DATA, DISSEMINATION & SPECIMENS

TRUSTEES OF BOSTON UNIVERSITY<br>By: ________<br>Name: ________<br>Title: ________<br>Date: ________<br>*Acknowledged by Site PI (Dr. Donald Thea):*<br>By: ________<br>Date: ________	THE INTERNATIONAL CENTER FOR DIARRHOEAL DISEASES RESEARCH, BANGLADESH (ICDDR,B)<br>By: ________<br>Name: ________<br>Title: ________<br>Date: ________<br>*Acknowledged by Site PI (Dr. Doli Goswami):*<br>By: ________<br>Date: ________
WITS HEALTH CONSORTIUM (PTY) LTD<br>By: ________<br>Name: ________<br>Title: ________<br>Date: ________<br>*Acknowledged by Site PI (Dr. Shabir Madhi):*<br>By: ________<br>Date: ________	THAILAND MINISTRY OF PUBLIC HEALTH – U.S. CENTERS FOR DISEASE CONTROL AND PREVENTION COLLABORATION<br>By: [signature]<br>Name: John R. MacArthur, MD, MPH<br>Title: Director<br>Date: 12 APR 2016<br>*Acknowledged by Site PI (Dr. Henry 'Kip' Baggett):*<br>By: [signature]<br>Date: 12 APR 2016

SIGNATURE PAGES
to the
PERCH AGREEMENT FOR DATA, DISSEMINATION & SPECIMENS

UNIVERSITY OF OTAGO,	CANTERBURY HEALTH LABS
A BODY CORPORATE ESTABLISHED UNDER THE UNIVERSITY OF OTAGO ORDINANCE 1869, THE UNIVERSITY OF OTAGO AMENDMENT ACT 1961 AND THE EDUCATION ACT 1989, OF DUNEDIN, NEW ZEALAND	
By: RBlaikie	By: ____________
Name: Professor Richard Blaikie ~~Deputy Vice-Chancellor, Research and Enterprise~~ University of Otago Dunedin	Name: ____________
Title: ____________	Title: ____________
Date: ~~12~~ 13 April 2016 RB	Date: ____________
Acknowledged by Core Team Secretariat Member – Clinical/Epidemiology (Dr. David Murdoch):	*Acknowledged by Core Team Secretariat Member – Laboratory (Dr. David Murdoch):*
By: DMurdoch	By: ____________
Date: APRIL 11, 2016	Date: ____________

SIGNATURE PAGES
to the

PERCH AGREEMENT FOR DATA, DISSEMINATION & SPECIMENS

UNIVERSITY OF OTAGO, *A BODY CORPORATE ESTABLISHED UNDER THE UNIVERSITY OF OTAGO ORDINANCE 1869, THE UNIVERSITY OF OTAGO AMENDMENT ACT 1961 AND THE EDUCATION ACT 1989, OF DUNEDIN, NEW ZEALAND*	CANTERBURY HEALTH LABS, Canterbury District Health Board (CDHB)
By: ______	By: [signature]
Name: ______	Name: Justine White
Title: ______	Title: GM Finance & Corporate Services
Date: ______	Date: 26/04/16
Acknowledged by Core Team Secretariat Member – Clinical/Epidemiology (Dr. David Murdoch):	*Acknowledged by Core Team Secretariat Member – Laboratory (Dr. David Murdoch):*
By: ______	By: [signature]
Date: ______	Date: 26/4/16

ANNEX A1

PERCH CONSORTIUM

DATA SHARING AGREEMENT

I. PREAMBLE

The PERCH Consortium Members recognize that in accordance with its charitable mission, the Bill & Melinda Gates Foundation (BMGF) is "committed to optimizing the use of health-related data to translate knowledge into life-saving interventions."

Accordingly, the PERCH Consortium Members are cognizant that in order to achieve that commitment, the PERCH Investigators must have a plan and a timetable to make all collective PERCH Data widely available to the global scientific and public health communities while also recognizing and respecting that the PERCH data were generated by the intellectual creativity, expertise, and efforts of the PERCH Consortium Members. Moreover, the PERCH Consortium must strive to do so at the earliest possible opportunity, using appropriate data access practices that foster broad and prompt dissemination to the scientific community.

The PERCH Consortium Members are aware that BMGF goals for wide access to the PERCH Datasets include:

(a) To promote collaboration between teams within and across institutions and across diverse disciplines, resulting in greater productivity and creativity.

(b) To encourage the testing of alternative hypotheses on pneumonia disease and its relation to young child mortality.

(c) To encourage meta-analyses.

(d) To help synthesize results in a broad context that may allow new insights and identification of actions and strategies that may save lives.

(e) To avoid duplication of efforts.

(f) To enable further analyses of existing PERCH Datasets.

(g) To help direct limited resources to the most promising research avenues and projects.

(h) To encourage independent analysis and verification of data or to provide new insights through alternative interpretations of the PERCH Datasets.

(i) To strengthen capacity and facilitate the training of junior investigators using the PERCH Datasets.

(j) To enable broader access to PERCH Datasets for secondary analysis, particularly for investigators in developing countries.

II. GUIDING PRINCIPLES OF THE PERCH CONSORTIUM DATA SHARING AGREEMENT

2.1. The processes and procedures for access to the PERCH Datasets will be consistent with the BMGF Global Access Objectives, and will be transparent and consistent with Good Clinical Data Management Practices.

2.2. All who have contributed to producing, sharing, and using PERCH Data must also commit to sharing responsibility for assuring that PERCH Data are accessed and used in ways that are consistent with applicable laws, regulations, and international standards of ethical research conduct.

2.3. The aim of benefiting the individuals and communities who enabled and supported the PERCH studies should be promoted to the furthest extent possible. This is of particular importance since the PERCH Project involved vulnerable individuals (children < 60 months of age) and their communities in developing countries.

2.4. PERCH Investigators adhere to the fundamental concept that sharing of scientific data from the PERCH Datasets will facilitate and accelerate the progress of research on pediatric pneumonia disease, child survival in resource scarce settings, and microbiologic information and will extend it in the following ways:

(a) Sharing provides a repository of knowledge and information upon which other researchers can build.

(b) Sharing broadens the scope of research.

(c) Sharing diversifies the scope of research by encouraging investigators from other disciplines, perspectives, and viewpoints to contribute their expertise.

(d) Sharing allows limited research resources to be used in a more efficient way by avoiding duplications of efforts.

(e) Sharing may lead to alternative interpretations of the same data or to modified analyses (e.g., based on future analytical methods that may be developed but that were not existent or widely accepted at the time of the initial analyses).

(f) Sharing increases the impact of the findings and results.

(g) Sharing can potentially diminish the number of research subjects required to answer questions by avoiding duplication of studies and instead analyzing relevant data from an existing dataset to answer the question.

(h) Sharing can stimulate the development of new analytical and biostatistical methods motivated by the desire and expertise of other investigators to mine the dataset for their own purposes, thereby providing a new tool that may be advantageous to many other groups and investigators working on the PERCH Project or other datasets.

(i) Sharing is also a potent discouragement to scientific misconduct which is otherwise a possibility when large numbers of clinical, laboratory and

epidemiologic investigators are involved in a multi-site complex project. Thus the transparency that accompanies data sharing enhances confidence in the data by the broad scientific as well as the lay community.

(j) Sharing encourages innovation and creativity that may come from individual scientists or groups of collaborating scientists outside the project who approach the data in fresh ways unfettered by the views of the initial analyzers of the data.

III. SPECIFIC ASPECTS OF THE PERCH DATA SHARING PLAN

3.1 <u>PERCH Datasets</u>

3.1.1 The PERCH Datasets include the PERCH Raw Datasets (i.e. complete set of raw datasets) and the PERCH Analytic Datasets (i.e. analytic datasets, which contain a subset of the raw variables and a set of calculated variables defined for analyses). The raw variables included in the analytic datasets represent those identified as key analytic variables that were more thoroughly reviewed in the data cleaning process. In most circumstances, the PERCH Analytic Datasets, appended with any additional variables identified as important for a given analysis, will be the only datasets shared across PERCH Consortium Members and with External Investigators.

3.1.2 The PERCH Datasets for each individual PERCH site will be made available to the respective Site PIs of the relevant site throughout the conduct of the study via the secure study website so the local PERCH Investigators can initiate site-specific analyses of the PERCH Data. The PERCH Core and Data Coordinating Center Members will have access to the data from all sites. The versions of the datasets made available throughout the study prior to the formal data freezes for the foundational and primary analyses represent ‘working versions’ for sites, PERCH Core, and Data Coordinating Center Members to begin analyses.

3.1.3 Upon completion of data collection, laboratory testing and data cleaning for the purposes of the primary and secondary analyses specified by the PERCH Project, the PERCH Datasets will be frozen at specified time-points. Separate data freezes will occur prior to completion of the foundational analyses and prior to completion of the analyses for the primary publication. The PI of each site will receive the frozen datasets for their respective site following each specified freeze.

3.1.4 Subsequent, sequential data freezes may occur following the freeze for the primary publication to incorporate additional laboratory testing data and/or to address data cleaning issues identified in the process of performing analyses. The PI of each site and any investigators engaged in data sharing will receive the frozen datasets following these subsequent data freezes as appropriate based on their analyses and the modifications made to the dataset.

3.1.5 The PERCH Datasets will respect the identity, confidentiality, and privacy of the individual subjects enrolled in the PERCH Project along with their families and the communities from whom data were collected.

3.1.6 Accompanying the PERCH Datasets, a code dictionary, explanation of terms, and other relevant data transfer materials will also be provided to all investigators who access the data to facilitate manipulation and analyses of the PERCH Datasets.

3.2 PERCH Consortium Analysis Period (PCAP)

3.2.1 Commencing from the date that PERCH Datasets are frozen for the primary publication, there will be a period referred to as the PERCH Consortium Analysis Period (PCAP) which will continue until March 31st 2019, or 36 months following the data freeze for the primary publication, whichever is later.

3.2.2 During this period, the focus of the PERCH Consortium will be to complete the core analyses for the multisite and site-specific output to address the primary and secondary objectives of the PERCH project.

3.2.3 Collaborative analyses with groups that are not part of the PERCH Consortium may be undertaken and are encouraged after the primary analyses are completed. The types of collaborations or new analyses during this period will be primarily for high priority strategic needs. Data access requests that are beyond the strategic priorities will be considered by the PERCH Data Access Committee during the PCAP, but are likely to be of secondary priority.

3.2.4 External Investigators or PERCH Consortium Members requesting data from sites they are not affiliated with will be required to submit a written proposal to the PERCH Data Access Committee articulating the analysis question, the proposed collaboration agreement, the proposed data to be accessed, and the proposed analytic approach. For PERCH Consortium Members, this requirement is specific to any newly submitted analyses and data access requests after the data freeze for the primary publication.

3.2.5 During this period, the intention will be for the PERCH Consortium to conduct the analysis if they elect to do so or, if the PERCH Consortium chooses not to undertake the analysis, to collaborate in analyses with External Investigators. Collaboration with the PERCH Consortium is a requirement for data access during the PCAP.

3.2.6 If data is to be shared with collaborators, an analytic dataset with any additional variables of interest included will be distributed, and the Raw Datasets will be held by PERCH Core and the PERCH sites.

3.2.7 The PERCH Data Access Committee is given the opportunity to indicate whether a proposed analysis is already completed or being undertaken and therefore decline data access.

3.2.8 The PERCH Consortium has the right for first publication of an analysis if the same analysis is performed by requestor.

3.2.9 The PERCH Data Access Committee has the right to prioritize requests for analyzed data.

3.2.10 Investigators will provide to the PERCH Data Access Committee full, transparent, acceptable documentation of the analysis itself (statistical coding, analysis plan, documentation of output) when possible.

3.2.11 The PERCH Executive Committee must be notified of all publications from the proposed analysis and collaboration and will review and provide input as needed, depending on the type of analysis (**see Annex 3**). PERCH Consortium Members will be included as a co-author as appropriate based on the type of analysis and collaboration involved. The PERCH Study Group is to be acknowledged in the manuscript, if not included as a co-author (**see Annex 3**).

3.2.12 During the PCAP, the PERCH Executive Committee and Core Team will have unique responsibility to coordinate access to the specific PERCH Datasets for composite (pan-site) analyses. Collaboration and sharing of PERCH Datasets across sites are strongly encouraged.

3.2.13 The PERCH sites are expected to collaborate to publish together. See the PERCH Consortium Publication Agreement (**Annex A3**).

3.3 <u>PERCH Extended Access Period (PEAP)</u>

3.3.1 Following the completion of the PCAP will be a second period lasting until March 31st 2024, or 60 months following the PCAP, whichever is later, termed the PERCH Extended Analysis Period (PEAP). During this period, the PERCH Consortium will serve primarily as a custodian of the data and oversee the processes of data access to ensure that data protections, including compliance with informed consent and ethical/regulatory requirements, are respected.

3.3.2 During the PEAP, collaboration with the PERCH Consortium is encouraged and offered but it is not a requirement for data access.

3.3.3 As during the PCAP, investigators will need to submit a written proposal articulating the analysis question, the proposed collaboration agreement, the proposed data to be accessed which must be reviewed and approved by the PERCH Data Access Committee prior to distributing the data.

3.3.4 Investigators conducting the analysis will provide to the PERCH Data Access Committee full, transparent, acceptable documentation of the analysis itself (statistical coding, analysis plan, documentation of output) once complete, when possible.

3.3.5 The PERCH Executive Committee must be notified of all publications from the proposed analysis and will review and provide input as needed, depending on the type of analysis (**see Annex 3**). PERCH Consortium Members will be included as a co-author as appropriate based on the type of analysis and collaboration involved. The PERCH Study Group will be acknowledged in any manuscript developed using data from the PERCH Project (**see Annex 3**).

3.4 <u>Accessing the PERCH Datasets</u>

3.4.1 The repository for the PERCH Datasets will be at IVAC or at IVAC's designated repository (e.g., the Data Coordination Center, The Emmes Corporation).

3.4.2 External Investigators who desire to access and analyze the PERCH Datasets or PERCH Consortium Members requesting data from sites with which they are not affiliated must complete a Dataset Access Request Form developed by the PERCH Consortium.

3.4.3 In this Dataset Access Request Form, the investigators will identify themselves and their institutions; provide contact information (preferably an email address); and submit a brief outline describing the purpose of their request to access the PERCH Datasets, including the analysis question, any proposed collaboration agreement, and the proposed data to be accessed.

3.4.4 The Dataset Access Request Form is not intended to be an impediment to gaining access to the PERCH Datasets. Rather it is meant to provide information about and allow tracking of the individuals and groups who access the PERCH Datasets and the purposes for which the PERCH Datasets will be used. This is also intended to facilitate collaborations among External Investigators and PERCH Investigators.

3.4.5 Investigators will be required to sign the Confidential Disclosure Agreement (in the form of **Exhibit B**.)

3.4.6 In reading and signing the Confidential Disclosure Agreement, investigators will acknowledge that they will not receive data with any personal identification codes, nor be provided with any link that might potentially identify the individual subjects enrolled in PERCH and their families and the communities from whom data were collected.

3.4.7 In reading and signing the Confidential Disclosure Agreement, the investigator will also be made aware of the contribution of the PERCH Consortium Members who worked for many years to generate the PERCH Data. The investigator must acknowledge that the PERCH Executive Committee must be notified of all publications resulting from the proposed analysis and collaboration and that the PERCH Executive Committee will review publications as needed to provide input, depending on the type of analysis (**see Annex 3**). The Investigator agrees to include PERCH Consortium Members as co-authors as appropriate based on the type of analysis and collaboration involved, and acknowledge The PERCH Study Group in the manuscript (**see Annex 3**).

3.4.8 In reading and signing the Confidential Disclosure Agreement, the investigator will acknowledge that anyone who uses the PERCH Datasets also becomes a steward to help maintain the integrity of those datasets. These investigators must accept that they are also responsible for using the PERCH Datasets in ways that are consistent with Good Clinical Data Management Practices, and with applicable laws, regulations, and international standards of ethical research conduct.

3.5 PERCH Data Access Committee

3.5.1 During the PCAP and PEAP, the PERCH Data Access Committee has responsibility for reviewing all Data Access Requests (Dataset Access Request Forms) submitted by External Investigators or PERCH Consortium Members requesting data from sites with which they are not affiliated.

3.5.2 The PERCH Data Access Committee will work through a consensus process. In the event an issue cannot be resolved through consensus building, the committee will resort

to voting on an issue. All members of the committee will be offered the opportunity to vote within a reasonable timeframe not to exceed thirty (30) calendar days. A simple majority will determine the outcome.

3.5.3 Data requests will be submitted to any site-level approval committees, as required by the site, prior to the transfer of data to the Recipient.

3.5.4 The PERCH Data Access Committee will also be responsible for determining the long-term storage and access to the PERCH Datasets following the completion of the PCAP and PEAP (i.e., the complete period lasting until March 31st 2024, or ninety-six (96) months following data freeze for the primary publication, whichever is later).

The PERCH Data Access Committee consists of:

a. The PERCH Executive Committee (10 members) and

b. A representative of the BMGF (1 member).

ANNEX A2

PERCH CONSORTIUM

MATERIAL SHARING AGREEMENT

I. PREAMBLE.

The PERCH Consortium Members recognize that in accordance with its charitable mission, the Bill & Melinda Gates Foundation ("BMGF") is "committed to optimizing the use of health-related data to translate knowledge into life-saving interventions." A salient feature of the PERCH Project is the collection and archiving of blood, urine, and respiratory specimens from both cases and controls from the PERCH sites in sub-Saharan Africa and Asia, consequent to the efforts of the PERCH Consortium.

The BMGF is eager for the PERCH Project to offer to the wider scientific and public health communities controlled access to these Materials so that investigators can perform hypothesis-driven research, confirmatory investigations, and follow-on studies. The PERCH Executive Committee and PERCH Investigators appreciate the importance of this concept and recognize that this must be accomplished with a sound specimen repository and access plan.

The PERCH Consortium Members are aware that the BMGF's goals of making the Materials available include:

(a) To generate further insights from study of these Materials that may save the lives of infants and young children residing in developing countries.

(b) To stimulate the broader scientific and public health community to consider innovative ways to utilize the unique specimen resources generated through the PERCH project.

(c) To pursue further hypotheses related to the PERCH Data.

(d) To encourage related independent studies to expand on PERCH insights and to verify PERCH findings.

(e) To provide vaccine developers, public health officials and other interested parties further insights on the etiology of severe and very severe pneumonia in infants and young children in the least developed countries.

(f) To improve access to well characterized respiratory pathogens that were detected and identified by well-standardized assays.

(g) To help improve the molecular authentication of respiratory pathogens.

(h) To manage the distribution and quality control of the Materials.

(i) To manage controlled access to the small volumes of PERCH body fluid specimens from pediatric subjects with severe and very severe pneumonia and from community control subjects without severe pneumonia. These specimens represent the most precious PERCH resource materials because only a limited supply exists and the specimen stock cannot be replenished or expanded. In contrast, specific bacterial pathogens can be propagated to expand and replenish the Material within the repository.

II. SPECIMEN REPOSITORY AGREEMENT

The PERCH Project is an historic, highly coordinated, multi-site, collaborative research project that involves many investigators and collaborators who worked interactively over multiple years to generate the PERCH Data and Materials. Without the productive, harmonious interaction of all elements of the PERCH, this enormous and complex study could not have been completed.

In order to provide access to Materials, there must exist an equitable and transparent process that is endorsed by all PERCH Executive Committee members and Site PIs for allowing distribution of the Materials.

The respiratory pathogens and limited amounts of body fluids and nucleic acid specimens from cases and controls enrolled in the PERCH Project constitute an extraordinary resource for the international community of investigators interested in pneumonia etiology and risk factors.

2.1 Organizational Structure and Approach

2.1.1 The PERCH Biorepository Specimen and Access Committee has responsibility for systematically evaluating requests for sharing of Materials. The members of that committee will determine whether each request for Materials is approved in full, approved contingent upon modification, or disapproved.

2.1.2 The PERCH Biorepository Specimen and Access Committee consists of:

a. The PERCH Executive Committee (10 members);

b. A representative from the Foundation (1 member);

2.1.3 Decisions of the PERCH Biorepository Specimen and Access Committee will be made by majority vote of those committee members in attendance, provided that a quorum is reached. For the purposes of this Agreement, a quorum will be achieved with the Coordinating PI, at least one of the other Core team members of the EC and at least 4 site PI members of the EC.

2.1.4 The PERCH Executive Committee has responsibility for preparing, codifying, and overseeing implementation of the policies of the PERCH Biorepository Specimen and Access regarding release of Materials.

2.1.5 Release of Materials is contingent upon final approval from the site, including local ethics and (where applicable) scientific committee(s), and receipt of transport permits (where applicable).

2.1.6 The guidelines described herein refer all Materials collected as part of the PERCH project, including those stored at the PERCH site laboratories as well as in the Central Repository maintained at the Canterbury Health Laboratories (CHL), Christchurch, New Zealand.

2.1.7 The PERCH Specimen Repository includes study materials centrally located at CHL as well as study materials stored at the PERCH research sites. The central repository will contain the following Materials collected from enrolled cases and controls from each of the sites:

(a) Pathogens isolated from blood, nasopharyngeal, induced sputum, pleural fluid, and lung aspirate specimens, with the exception of *Mycobacterium tuberculosis*.

(b) Whole blood, sera, nasopharyngeal, induced sputum, and urine specimens.

(c) Additional material, such as extracted nucleic acid used in PCR assays.

All study materials not shipped to CHL will be maintained locally at the PERCH research sites. Sites may also request samples to be returned from the central repository for local storage.

2.1.8 Study materials remaining at the sites, though not part of the PERCH Central Repository, will be governed by this Agreement in the same manner as those that are part of the Central Repository.

2.1.9 The guidelines for sharing differ according to the nature of the Material requested.

(a) Guidelines that govern sharing of bacterial isolates take into consideration the fact that bacterial isolates are largely renewable resources potentially available in unrestricted quantity (subject to the caveat that during long-term storage in a frozen state some isolates may be subject to loss of their viability). An expedited review process will be used for requests of fewer than 100 bacterial isolates only (i.e., requests not including body fluid specimens). Members of the PERCH consortium wishing to access pathogen isolates stored at their own research laboratories will only need to provide notification to the Executive Committee and will not be required to submit an Application for Materials.

(b) Guidelines which govern body fluid and nucleic acid allocation respect the unique and precious nature of human specimens which are available in small, finite quantities. A full review is required for requests for body fluid material, nucleic acid, and requests for 100 or more bacterial isolates (because of the work involved in preparing the specimens to be shipped).

Note: any request that meets the criteria for an expedited review may be subject to a full review at the discretion of the reviewers based on the proposed research or analysis. Regardless of the type of review (full vs. expedited), any final decision for specimen access will be made by appropriate site review committees.

2.1.10 The PERCH Biorepository Specimen and Access Committee will report to the BMGF regarding requests for specimens, approvals, and non-approvals (together with the Committee's rationale for non-approval).

2.2 How to Apply

2.2.1 Applications for Materials by investigators, including External Investigators and PERCH Investigators, requesting specimens, will be processed by the PERCH Biorepository Access Committee.

2.2.2 When requesting Materials:

(a) Investigators must provide a rationale for access that involves a highly innovative research strategy and must also provide assurance that they have sufficient resources, expertise, and funding to complete the research proposed.

(b) Investigators must ask for the smallest amount (both volume and number) needed to perform scientifically meaningful experiments. New research techniques or new areas of investigation should be proposed as a pilot study in order to minimize the use of specimens for risky, unproven approaches. It may be desirable or necessary to modify study designs or utilize different materials to address the question where availability is limited.

2.2.3 In order to access Materials investigators must complete and submit the following documents electronically:

2.2.3.1 Prior to review by the PERCH Biorepository Specimen and Access Committee:

(a) PERCH Ancillary Study and/or Collaboration Request Application

2.2.3.2 Following review and approval by the PERCH Biorepository Specimen and Access Committee of the Application:

(a) PERCH Material Transfer Agreement (see **Exhibit A**)

2.3 PERCH Ancillary Study and/or Collaboration Request Application

2.3.1 The PERCH Ancillary Study and/or Collaboration Request Application will be made available to investigators who would like to request specimens.

2.3.2 The first section of the document is to be completed by the investigator to provide the PERCH Biorepository Specimen and Access Committee the investigator's identifying information (name, address, position, institutional affiliation, contact information [preferably an email address]). The investigator's *curriculum vitae* must be uploaded as an attachment.

2.3.3 Investigators must provide a description of the research to be performed with the accessed Materials with sufficient detail for the PERCH Biorepository Specimen and Access Committee to be able to weigh the scientific innovation and public health importance of the proposed research and to assess whether the investigators have the ability and resources to accomplish their stated goals according to the criteria described below.

2.3.4 This information will allow the PERCH Biorepository Specimen and Access Committee to track the individuals and groups who desire access to the Materials and the type of research that is proposed and to triage the application to the appropriate review process (i.e., expedited versus full review).

2.4 PERCH Material Transfer Agreement

2.4.1 The Investigator and the Investigator's Institution must sign the PERCH Material Transfer Agreement (**Exhibit A**) to receive Materials. By signing this agreement, the Investigator and Institution agree to the terms and conditions therein regarding the use of the Materials. Investigators will also agree to pay for the cost of shipping and any costs related to the preparation of specimens and/or isolates shipments, unless separate arrangements have been made.

2.5 Review Process for Requests involving Bacterial isolates only

2.5.1 Requests for 100 or fewer bacterial isolates only may be reviewed by the Coordinating PI or her designee, without full review by the PERCH Biorepository Specimen and Access Committee. The Coordinating PI reserves the right to request a full committee review if she deems it necessary.

2.5.2 A determination will be provided to the applicant no later than 1 month following the request if an expedited review is possible and no later than 2 months following the request if the full committee is reviewing.

2.6 Full Review Process / PERCH Biorepository Specimen and Access Committee:

2.6.1 The PERCH Biorepository Specimen and Access Committee will be responsible for reviewing all applications requesting body fluids, and/or nucleic acid, or requests for more than 100 bacterial isolates.

2.6.2 A quorum will be reached if the following are present: Coordinating PI, at least one of the other Core team members of the EC and at least 4 site PIs.

2.6.3 The PERCH Biorepository Specimen and Access Committee will convene as needed (approximately 4 times per year), generally via teleconference, to review requests for access to Materials. Members unable to attend may submit their review and recommendations in writing.

2.6.4 Investigators can expect a determination no later than 2 months after the request. Expedited review can be requested by an applicant if the research is considered to be urgent in nature. If the Coordinating PI concurs, an attempt will be made to convene the PERCH Biorepository Specimen and Access Committee expeditiously.

2.6.5 The following standardized set of criteria will be used to evaluate each application based on its scientific merit, innovation, scientific impact, strength of investigators, institutional and financial support, and ethical, safety and regulatory oversight:

(a) **Overall impact:** Will the proposed study have a strong positive impact on PERCH's ability to meet its primary objectives and/or carry no risk to the project?

(b) **Benefit to PERCH**: Does the study provide training, internship or publication opportunities, and in particular are these opportunities provided to young and/or local investigators? Does the proposed study have high public health importance? Strategic considerations with regards to partnerships and sponsors are also included here.

(c) **Scientific Merit:** Does the proposal address an important problem or critical barrier to progress in the field? Is PERCH well suited to answer these questions, and is the design likely to provide a conclusive answer?

(d) **PERCH's unique ability to answer the question:** Could other sources of data or specimens be used instead?

(e) **Investigators:** Are the investigators well suited to the project? If early stage investigators, do they have appropriate experience, training and mentoring? If the Investigators are more advanced in their careers, have they demonstrated an ongoing record of accomplishments in their field(s)?

(f) **Resources and environment:** Do the investigators have access to financial, human, and physical resources and institutional support necessary for successful completion of the proposed studies?

(g) **Innovation:** Does the application utilize novel approaches likely to advance the field?

(h) **Approach:** Are the overall strategy, methodology, and analyses well-reasoned and appropriate to accomplish the specific aims of the project? Are potential problems, alternative strategies, and benchmarks for success presented? If the project is in the early stages of development, will the strategy establish feasibility and will particularly risky aspects be managed?

(i) **Ethical oversight:** Are procedures in place to ensure that the research is performed in compliance with local ethical, veterinary, biosafety, regulatory, and other agencies, as appropriate?

2.7 Local Institutional Review Board and Ethics Committee Approvals for use of Material

2.7.4 Following study approval by the PERCH Biorepository Specimen and Access Committee the applicant must communicate with the principal investigator of each site from which materials are requested to determine whether additional Institutional Review Board or Ethics Committee approvals are needed prior to the transfer of materials.

ANNEX A3

PERCH CONSORTIUM

PUBLICATION AGREEMENT

I. PREAMBLE.

The PERCH Consortium Members recognize that in accordance with its charitable mission, the Bill & Melinda Gates Foundation is "committed to optimizing the use of health-related data to translate knowledge into life-saving interventions." Accordingly, the PERCH Consortium Members recognize that in order to achieve that commitment, the PERCH Data Access Committee must have a plan and a timetable to make collective data widely available (and at the earliest possible opportunity) to the wider scientific and public health communities through publications in peer review journals and presentations at national and international meetings.

The PERCH Consortium Members also fully recognize that this must be accomplished with a sound publication plan. The PERCH Consortium Members are aware that some of the goals of wide publication of data include:

(a) To describe in detail the clinical, epidemiological and microbiological methods used in the PERCH Project.

(b) To synthesize results of the PERCH Project in a broad context to identify new insights that may save the lives of infants and young children residing in developing countries.

(c) To stimulate responses among the scientific and public health community to results of the PERCH Project.

(d) To elicit further hypotheses related to the PERCH Datasets.

(e) To help direct limited resources towards the most promising research avenues and projects.

(f) To encourage related independent studies to expand on PERCH insights and to verify PERCH findings

(g) To encourage alternative innovative interpretations of the PERCH Datasets.

(h) To strengthen capacity and facilitate the training of new investigators in the writing of scientific papers for peer review.

(i) To enable broader dissemination of the results of the PERCH Project, particularly for investigators in developing countries.

(j) To provide vaccine developers, public health officials and other interested parties comprehensive information on the etiology of pneumonia in infants and young children in the least developed countries and on the adverse clinical consequences that follow pneumonia to assist in the design of vaccine development and implementation activities.

II. PUBLICATION AGREEMENT

The PERCH Project is an historic, highly coordinated, multi-site, collaborative research project that involves many investigators and collaborators who worked interactively over multiple years to generate the PERCH Data. Without the productive, harmonious interaction of all elements of the PERCH Project, this enormous and complex study could not have been completed. In order to disseminate the results of PERCH, there must exist an equitable and transparent process that is endorsed by all PERCH Executive Committee members and Core leaders for assigning authorship to publications.

The PERCH Data will constitute an extraordinary resource for the international community of investigators interested in pneumonia, respiratory pathogens and the reduction of young child mortality.

The PERCH Executive Committee has established the following principles that will govern authorship and publication rights.

(a) The PERCH Executive Committee will be charged with developing and implementing publications policy governance. This group will be composed of three PERCH Core members (Coordinating PI and two Co-PIs) and all seven Field Site PIs, and will be responsible for protecting the interests of all stakeholders in the writing and publication of study information.

(b) The findings from PERCH primary objectives from all seven study sites will be published before any individual sites publish their results. Individual site publications can be submitted immediately upon the pan-site manuscripts being accepted for publication by peer review journals.

(c) Authorship assignments and responsibilities will be established and defined for all investigators including the PERCH Executive Committee, other senior site investigators, the PERCH Core, Data Coordinating Members, biostatisticians and appropriate collaborators.

(d) The PERCH Executive Committee will establish *a priori* lists of potential investigations that are anticipated from the outset so that the expectations for these publications can be established as soon as possible.

(e) The PERCH Executive Committee encourages the development of junior investigators, and is supportive of this aim. The responsibility for their development falls within the responsibilities of the site PIs (site, in this instance, includes the PERCH Core).

(f) The PERCH Executive Committee will establish time limits for exclusivity of a proposed publication concept. After these established periods have expired, the initial allocations of responsibility or authorship may be revised if the obligations of exclusivity were not met.

III. KEY ELEMENTS OF PERCH PUBLICATION POLICY/PROCEDURES

3.1 The PERCH Executive Committee and PERCH Core will propose the list of site-specific and pan-site publications to address the primary and secondary objectives of the PERCH Project.

3.2 PERCH Investigators and External Investigators will develop ideas for additional publications and will complete a written proposal according to the principles set forth in **Annex 1**.

3.3 The PERCH Executive Committee will review the written proposals for all analyses and publications to:

(a) Confirm the type of analysis (single vs. multi-site) and recommend an authorship level.

(b) Consider whether the governing principles are being applied appropriately.

(c) Approve the analysis plan or request modifications as needed.

(d) Determine whether notification/input is required for the manuscript.

3.4 All PERCH Executive Committee Members will be notified of the status of each proposed publication at least twice per year.

IV. DEFINITION OF AN "AUTHOR"

The PERCH Executive Committee will define the contributions of authors according to the "Uniform Requirements for Manuscripts Submitted to Biomedical Journals," promulgated by the International Committee of Medical Journal Editors, as they may be amended from time to time. These are available at http://www.icmje.org.

V. AUTHORSHIP ROLES AND PLANNED PUBLICATIONS

The PERCH publications policy aims to help guide all stakeholders through the publication process for publications that will utilize and disseminate PERCH Data. This project will involve a number of interests, and definitions contained in this publications policy will need to balance the needs of the PERCH PIs, the other local PERCH Investigators, and the opportunity for young investigators to obtain lead authorship on ancillary studies. This publications policy utilizes as a guide the authorship groupings adapted from the "Multicenter AIDS Cohort Study Publication/Publicity Policy" (September 17, 2008) and the "Global Enterics Multi-Center Study (GEMS) Publication Agreement (November 1, 2011)".

There are three main types of publications anticipated from the PERCH Project, each with different considerations for authorship and review by the PERCH Executive Committee:

Authorship Level	Paper type	Recommended authors	Recommended acknowledgments	EC Input or Notification
1	Main paper(s)<br>(Primary etiology and primary risk factor papers)	PERCH Study Group	• Site contributors<br>• Pneumonia Experts Group (PEG)<br>• Emmes<br>• Canterbury Health Laboratories (where applicable)	• Input
2	Foundational DAPs[1] or other DAPs or substudies with all PERCH sites	PERCH EC and Core Team leads, Core team and 2 co-authors from each site (approx. 30 authors), Emmes (where applicable), PERCH Study Group	• PERCH Study Group (if not included in authors)<br>• PEG<br>• Emmes (where applicable)<br>• Canterbury Health Laboratories (where applicable)	• Input
3	Site-specific	Analysis team (including Core and Site team members where applicable), PERCH Study Group (where applicable) and Emmes (where applicable)	• PERCH Study Group if not an author<br>• PEG<br>• Emmes (where applicable)<br>• Canterbury Health Laboratories (where applicable)	• Notification
	Cross-site but not all PERCH sites			• Input
	Sub-study (excluding those involving data from all PERCH sites)			• Input (if uses data from 2+ sites); Notification if only uses data from 1 site
	Technical / methodological	Analysis team (including Core and Site team members where applicable) and Emmes (if applicable)	• PERCH Study Group<br>• Emmes (where applicable)	• Notification

[1]Data Analysis Plan (DAP)

These guidelines related to authorship were developed primarily for the publications prepared by PERCH Consortium Members. For External Investigators collaborating with the PERCH Consortium, any PERCH Consortium Members meaningfully engaged in the collaboration should be included as co-authors, where appropriate. For any publications developed using

PERCH data, “The PERCH Study Group” will be acknowledged, regardless of collaboration with the PERCH Consortium.

For additional details regarding authorship, including authorship order, recognition of other individuals who assisted in data collection (e.g., the chest x-ray reading panel), mentorship as related to authorship, the manuscript review process and the complete list of PERCH Study Group Members and PERCH Study Contributors, see the “PERCH Publication and Authorship Guidelines”.

The following funding language should be included in all manuscripts: “The PERCH Project was supported by grant 48968 from The Bill & Melinda Gates Foundation to the International Vaccine Access Center, Department of International Health, Johns Hopkins Bloomberg School of Public Health.”

VI. REVIEW BY CORE TEAM AND EXECUTIVE COMMITEE

All manuscripts relating to the PERCH Project must be reviewed by the PERCH Core Team at least twenty-one (21) days before submission for publication. Any abstracts relating to the PERCH Project must be reviewed by the PERCH Core Team at least fourteen (14) days before submission. Draft manuscripts will be submitted to the PERCH Core Team with the first section of the “Manuscript Review Form” completed. The PERCH Core Team will be responsible for circulating the manuscript to the PERCH Executive Committee and compiling comments for the author. The author shall reasonably consider any comments made by the PERCH Executive Committee. For manuscripts requiring PERCH Executive Committee input, a revised version of the manuscript incorporating any necessary comments should be submitted to the PERCH Core Team prior to submission to the journal. A final copy of the publication will be sent to the PERCH Core Team. See PERCH SOP 4.0 PERCH Manuscript Review and Manuscript Review Form for additional details.

EXHIBIT A

PERCH CONSORTIUM

MATERIAL TRANSFER AGREEMENT

This Material Transfer Agreement is made as of _______________ (the **"Effective Date"**) between [**INSERT NAME OF PERCH CONSORTIUM MEMBER**] ("**Provider**,") and **[INSERT NAME OF EXTERNAL INVESTIGATOR'S INSTITUTION/OTHER PERCH CONSORTIUM MEMBER(S)' INSTITUTION**] on behalf of **[INSERT NAME AND ROLE OF THE INVESTIGATOR AT THE EXTERNAL INSTITUTION/OTHER PERCH CONSORTIUM INSTITUTION]** ("**Recipient**").

BACKGROUND

WHEREAS, Provider is a member of the Pneumonia Etiology Research for Child Health (the **"PERCH Consortium"**), which is a network of research institutes, centers and academic units created to collaborate in the implementation of the scientific research plan of the PERCH project, namely the creation of knowledge on the etiology of severe and very severe pneumonia among infants and young children in developing countries, in order to provide a basis for the sound implementation of existing interventions and treatments and investment in the highest priority needed new interventions (the **"PERCH Project"**).

WHEREAS, the PERCH Project is funded by a grant from the Bill & Melinda Gates Foundation (the **"Foundation"**) to The Johns Hopkins Bloomberg School of Health pursuant to a grant entitled "Pneumonia Etiology Research for Child Health Project."

WHEREAS, Provider possesses certain respiratory pathogens, body fluids and other materials accumulated in the course of the PERCH Project, and which, along with any derivatives created therefrom by Recipient, constitute the "**Materials**."

WHEREAS, Recipient has submitted a request to use the Materials for conducting the research described in the "**PERCH Ancillary Study and/or Collaboration Request Application"**, and such request has been approved by the PERCH Biorepository Specimen and Access Committee.

Now, therefore, the parties agree to the following terms:

<u>DEFINITIONS:</u>

"Global Access Objectives" means (i) the broad and prompt dissemination of research information generated through use of the Materials to the scientific community and (ii) the development of a vaccine through use of the Materials that will be made accessible to the people most in need in the developing world in its use of the Materials and any improvements, modifications, or inventions that may arise through such use.

"Commercial Purposes" means the use or transfer of Materials, PERCH Data, or Confidential Information by, to, on behalf of, or for research sponsored by a for-profit company.

"Modifications" means any modification which contains or incorporates any of the Materials.

"**Research**" means the research described in the PERCH Ancillary Study and/or Collaboration Request Application submitted to and approved by the PERCH Biorepository Specimen and Access Committee.

TERMS AND CONDITIONS OF THIS AGREEMENT:

1. The Parties acknowledge that the approved <INSERT SPECIMEN REQUEST STUDY TITLE NAME> is hereby incorporated by reference as a part of this Agreement and shall be attached as "Exhibit A" hereto.
2. Recipient agrees that the Materials are to be used solely for the Research and not beyond the duration described in the PERCH Ancillary Study and/or Collaboration Request Application.
3. Recipient shall use the Materials in accordance with safe laboratory practices and the highest standards of skill and care. Recipient shall ensure compliance with any applicable laws and regulations governing the transportation, keeping or use of the Materials.
4. Materials are to be used only in the Recipient Investigator's laboratories, and only under the direction of authorized personnel.
5. Materials will not be given or made available to any third party unless approval to do so has been granted in writing by Provider or the PERCH Biorepository Specimen and Access Committee. If such approval is granted, any permitted transfer must also be subject to the conditions of this Agreement. Recipient will refer any third party to the PERCH Biorepository Specimen and Access Committee.
6. Provider retains ownership of the Materials and any Modifications. Except as specifically set forth in this Agreement, no expressed or implied license or other rights are provided to Recipient under any proprietary rights of Provider.
7. Recipient agrees and acknowledges that:

a. Materials will not be used in human subjects, in clinical trials, or for diagnostic purposes involving human subjects unless such use is expressly approved by Provider in writing, and Recipient's use shall be in accordance with the relevant clinical protocol, informed consent and subject to any required Institutional Review Board and/or ethics review committee approvals and/or other necessary approvals as applicable;

b. Materials will only be used by individuals who are legally obligated, in the manner and to the extent required in the applicable PERCH Ancillary Study and/or Collaboration Request Application, to allocate their respective right in any and all inventions (and any patent rights or other rights arising therefrom);

c. Recipient acknowledges that the Materials were generated through the use of funding by the Bill & Melinda Gates Foundation;

d. Recipient will adhere to the Global Access Objectives;

e. Materials are provided without personal identification codes;

f. Recipient accepts responsibility to respect the identity, confidentiality, and privacy of the individual research subjects enrolled in the PERCH Project who provided the relevant samples, and to similarly respect the families of these subjects and the communities where they reside;

g. The PERCH Consortium worked for many years to generate the Materials and to manage and store them;

h. If required by applicable law or regulation of the country from which the Materials originated, Recipient must return or destroy any remaining Materials after a period of thirty six (36) months after receipt. However, this period may be extended for an additional thirty six (36) months upon written request by the Recipient and consideration by the PERCH Biorepository Specimen and Access Committee.

i. Unless specifically set forth in this Agreement, Recipient shall not be entitled to use the Material provided by the Provider for Commercial Purposes without separate written agreement to that effect.

j. Acknowledge the PERCH Study Group in any manuscripts developed from the Research as set forth in the PERCH Publication Agreement. In addition, the following funding language should be included in all manuscripts: “This PERCH Project was supported by grant 48968 from The Bill & Melinda Gates Foundation to the International Vaccine Access Center, Department of International Health, Johns Hopkins Bloomberg School of Public Health.”

k. Submit any manuscripts developed from the Research to the PERCH Executive Committee for review and input as set forth in the PERCH Publication Agreement.

8. It is acknowledged that the results of the Research using the Materials may be important to Provider in its attempts to attract good researchers and secure research funding for its research. Such recognition may be primarily established by reference to the use of Materials by third parties, such as Recipient, in publications. It is further acknowledged that the failure to obtain such recognition may adversely affect Provider’s ongoing research activities and funding. Accordingly, Recipient agrees that it will notify Provider and, at least 21 days prior to submission, provide a copy of any Publication concerning the Research to Provider. Recipient shall reasonably consider any comments Provider offers and will make appropriate attributions (co-authorship or acknowledgement) in all such publications where Provider’s Materials were used in Recipient’s Research. Recognition for the contribution of Provider should be established by acknowledging use of the Materials by Recipient in any such publication.

9. Recipient will inform Provider of results of the Research, with a written report of the results within sixty (60) days after conclusion of the Research.

10. Any Materials transferred pursuant to this Agreement are understood to be experimental in nature and may have hazardous properties. Provider makes no representations nor extends any warranties of any kind, either expressed or implied. There are no express or implied warranties of merchantability or fitness for a particular purpose, or that the use of the materials will not infringe any patent,

copyright, trademark, or other third party proprietary rights, or that the materials will not pose a health or safety risk.

11. Recipient shall pay Provider for any reasonable shipping and related costs that may be incurred when preparing and sending the Materials to Recipient which shall be made apparent by Provider to Recipient prior to the incursion such costs. Payment shall be in made in the manner indicated on the PERCH Ancillary Study and/or Collaboration Request Application.

12. All Materials will be shipped as agreed upon by Provider and Recipient (see Exhibit A).

13. Except to the extent prohibited or, where applicable, to the extent authorized by law, Recipient assumes liability for claims for damages to the extent caused by its use, storage, and/or disposal of the Materials for activities carried out pursuant to this Agreement. Provider will not be liable to Recipient for any loss, claim, or demand made by Recipient, or made against Recipient by any other party, to the extent caused by the use, storage, and/or disposal of the Materials by Recipient, except to the extent permitted by applicable law when such loss, claim, or demand is caused by the gross negligence and/or willful misconduct of Provider.

    The University of Maryland, its officers and employees acting within the scope of their employment by the University of Maryland are subject to the Maryland Tort Claims Act ("the Act'), Title 12, Subtitle 1, State Government Article, Annotated Code of Maryland, which permits claims in tort against the State of Maryland under certain circumstances. In order to file a claim under the Act, a claimant must submit a written claim to the Treasurer of the State of Maryland or a designee of that office within one year after the injury to the person or property that is the basis for the claim.

14. Recipient agrees to handle, store, and use the Materials in a safe manner and in compliance with all applicable statutes and regulations, including applicable governmental regulations and guidelines as well as the requirements of national drug regulatory authorities and other relevant regulatory agencies.

15. Recipient acknowledges that approval by the PERCH Biorepository Specimen and Access Committee does not confer approval for use of the specimens by the PERCH sites' Institutional Review Board and/or ethics review committees and that these approvals must be obtained prior to the shipment of materials.

16. Recipient agrees that it shall obtain any Institutional Review Board and/or ethics review committee and/or other approvals that may be required for the use of Materials received under this Agreement as outlined in the respective PERCH Ancillary Study and/or Collaboration Request Application prior to using such Materials in its research conducted under this Agreement.

17. This Agreement will terminate upon completion of the Research by Recipient or five (5) years from date of the last party's signature, whichever occurs first. At that time, Recipient will discontinue use of the Materials and will promptly give written notice to Provider. The Provider may, at its option, direct Recipient to either:

a. Destroy any remaining Materials and Modifications, and to confirm in writing that destruction; or

b. Return any remaining Materials and Modifications. The cost of shipment will be at Recipient's expense.

18. Expiration or termination of this Agreement does not relieve either party of any obligation which arises before expiration or termination, including without limitation obligations for payment and reporting. Any provision of this Agreement which contemplates performance or observance subsequent to any termination or expiration of this Agreement shall survive any termination or expiration of this Agreement and continue in full force and effect.

19. **Dispute Resolution.** All disputes under this Agreement shall be resolved and conducted, regardless of the means or authority, in the English language. Any dispute or controversy arising in connection with this Agreement shall first be referred to the parties' respective officers that signed this document, on behalf of the Parties, or their successors, for attempted resolution in good faith negotiations within sixty (60) days of notice of such dispute. If such officers are not able to resolve the dispute within the sixty (60) day period, or any agreed upon extensions, the Parties shall be free to resolve the dispute through any dispute resolution mechanism they may individually or collectively choose. Each party agrees that, prior to resorting to litigation to resolve any dispute, it will confer with the other party to determine whether other procedures that are less expensive or less time consuming can be adopted to resolve the dispute.

SIGNATURE PAGE TO FOLLOW

IN WITNESS of this Agreement, the parties have executed this Agreement through their duly authorized representatives.

**Provider**	**Recipient**
By: ______________________	By:______________________
Name: ______________________	Name______________________
Title:______________________	Title:______________________
Date: ______________________	Date: ______________________

The Recipient PI and Providing PI, by affixing their signatures below, acknowledge that they have read, understood, and agree to comply with the terms of this Agreement.

**Provider PI**	**Recipient PI**
By: ______________________	By:______________________
Name: ______________________	Name______________________
Title:______________________	Title:______________________
Date: ______________________	Date: ______________________

EXHIBIT B

PERCH CONSORTIUM
CONFIDENTIAL DISCLOSURE AGREEMENT

This Confidential Disclosure Agreement ("CDA") is made as of ___________ (the **"Effective Date"**) between [**INSERT NAME OF PERCH CONSORTIUM MEMBER**] ("**Provider**,") and **[INSERT NAME OF EXTERNAL INVESTIGATOR'S INSTITUTION/OTHER PERCH CONSORTIUM MEMBER(S)' INSTITUTION**] on behalf of **[INSERT NAME AND ROLE OF THE INVESTIGATOR AT THE EXTERNAL INSTITUTION/OTHER PERCH CONSORTIUM INSTITUTION]** ("**Recipient**").

BACKGROUND

WHEREAS, Provider is a member of the Pneumonia Etiology Research for Child Health Project (the **"PERCH Consortium"**), which is a network of research institutes, centers, and academic units created to collaborate in the implementation of the scientific research plan of the PERCH Project, namely the creation of knowledge on the etiology and burden of pediatric pneumonia disease among infants and young children in developing countries, in order to provide a basis for the sound implementation of existing interventions and treatments and prioritizing new investments (the **"PERCH Project"**).

WHEREAS, the PERCH Project is funded by a grant from the Bill & Melinda Gates Foundation (the **"Foundation"**) to The Johns Hopkins University pursuant to a grant entitled "Pneumonia Etiology Research for Child Health Project."

WHEREAS, Provider possesses certain data used or generated in the performance of the PERCH Project (**"PERCH Data"**).

WHEREAS, Recipient has submitted a request to use the data for conducting the research described in the "**Dataset Access Request Form"** to the PERCH Data Access Committee (the "**Research**").

Now, therefore, the parties agree to the following terms:

DEFINITIONS:

"Affiliate" means any business entity controlled by, controlling or under common control of a person. Such control shall include beneficial ownership of more than fifty percent (50%) of the voting interest in an entity, or such other relationship as, in fact, constitutes actual control.

Commercial Purposes" means the use or transfer of Materials, PERCH Data, or Confidential Information by, to, on behalf of, or for research sponsored by a for-profit company.

"Confidential Information" means, subject to Section 4, all PERCH Data and other proprietary and non-public information with respect to any aspect of a PERCH Project, if not identified or marked as confidential at the time of disclosure, a reasonable person under the circumstances would deem to be confidential.

TERMS AND CONDITIONS OF THIS AGREEMENT

1. The Parties acknowledge that the approved <INSERT DATA REQUEST STUDY TITLE NAME> is hereby incorporated by reference as a part of this Agreement and shall be attached as “Exhibit A” hereto.
2. Each Recipient agrees that it will:
    a. Use the Confidential Information received from a Provider solely for the Research,
    b. Treat the Confidential Information with reasonable care to avoid disclosure of the Confidential Information to any third party, person, firm or corporation other than as expressly stated herein, and
    c. Except to the extent prohibited or, where applicable, to the extent authorized by law, be responsible for use of the Provider's Confidential Information outside the scope of the Research as well as for any unauthorized disclosure directly resulting from their negligent or willful failure to exercise such reasonable care
    d. Acknowledge the PERCH Study Group in any manuscripts developed from the Research as set forth in the PERCH Publication Agreement. In addition, the following funding language should be included in all manuscripts: “The PERCH Project was supported by grant 48968 from The Bill & Melinda Gates Foundation to the International Vaccine Access Center, Department of International Health, Johns Hopkins Bloomberg School of Public Health.”
    e. Submit any manuscripts developed from the Research to the PERCH Executive Committee for review and input as set forth in the PERCH Publication Agreement.
3. Each Recipient acknowledges that:
    a. The PERCH Consortium has worked for many years to generate the PERCH data.
    b. No data will be received that contains any personal identification codes, nor be provided with any link that might potentially identify the individual subjects enrolled in PERCH and their families and the communities from whom data were collected.
    c. Recipient will help maintain the integrity of PERCH datasets and will be responsible for using the PERCH datasets in ways that are consistent with Good Clinical Data Management Practices, and with applicable laws, regulations, and other applicable standards of ethical research conduct.
    d. Data requests will be submitted to any site-level approval committees, as required by the site, prior to the transfer of data to the Recipient.
4. Notwithstanding anything to the contrary in this CDA, the Recipient shall have no obligation with respect to the Confidential Information received from a Provider to the extent such information is:
    a. Already known by the Recipient at the time of disclosure as can be demonstrated by contemporaneous documentation;

b. Publicly known, or subsequently becomes publicly known, without the wrongful act or breach of this CDA by the Recipient;

c. Rightfully received by the Recipient from a third party having the lawful right to make such a disclosure, where said disclosure is rightfully made without an express obligation of confidence;

d. Approved for release or disclosure by written authorization of the Provider;

e. Independently developed by the employees or agents of the Recipient without the use or knowledge of the Confidential Information provided by the Provider as can be demonstrated by contemporaneous documentation; or

f. Required to be disclosed pursuant to any competent judicial or government request, requirement or order, provided that the Recipient so disclosing takes reasonable steps to provide the Provider with sufficient prior notice in order to allow the Provider to contest such request, requirement or order and provided that such Confidential Information is disclosed only subject to reasonably available restrictions on further disclosure and use, and otherwise remains subject to the obligations of confidentiality and restricted use set forth in this CDA.

5. Each Recipient shall be entitled to disclose the Provider's Confidential Information to its employees and the employees of its Affiliates as well as its agents and consultants who are made aware of and will comply with the confidentiality and restricted use obligations no less strict than those set out herein. However, each Recipient shall only disclose the Provider's Confidential Information to those of its employees, agents, consultants and Affiliates who shall reasonably need to know such Confidential Information in order to evaluate such Confidential Information for the Research and/or to make decisions or render advice in connection with the Research and who shall be informed of the existence of this CDA and shall agree in writing or are subject to Recipient’s employment policy to comply with the terms hereof or be otherwise bound by law not to disclose such Confidential Information. Each Recipient shall be responsible for ensuring that its employees, agents and consultants of its Affiliates, and its consultants who receive Confidential Information comply with the terms of this CDA.

6. Subject to exemptions and limitations elsewhere in this CDA, the obligations of this Agreement shall remain in effect for each subject disclosure of Confidential Information during the Term of this Agreement and for a period of five (5) years from date of the execution of this agreement.

7. Unless specifically set forth in this Agreement, no rights in the Confidential Information are provided under any patent applications, patents, or other proprietary rights of the Provider. Unless specifically set forth in this Agreement, Recipient shall not be entitled to use the Confidential Information provided by the Provider for Commercial Purposes without separate written agreement to that effect.

8. The Recipient agrees to discontinue its use of the Confidential Information and destroy or return to the Provider all written Confidential Information received hereunder or Confidential Information that has been reduced to a written form upon completion of its use in accordance with this CDA or upon request by the Provider (which ever shall occur first); *provided, however,* one (1) copy of such Confidential Information may be retained by the Recipient to preserve an archival record of the same.

9. **Dispute Resolution.** All disputes under this Agreement shall be resolved and conducted, regardless of the means or authority, in the English language. Any dispute or controversy arising in connection with this Agreement shall first be referred to the parties' respective officers that signed this document, on behalf of the Parties, or their successors, for attempted resolution in good faith negotiations within sixty (60) days of notice of such dispute. If such officers are not able to resolve the dispute within the sixty (60) day period, or any agreed upon extensions, the Parties shall be free to resolve the dispute through any dispute resolution mechanism they may individually or collectively choose. Each party agrees that, prior to resorting to litigation to resolve any dispute, it will confer with the other party to determine whether other procedures that are less expensive or less time consuming can be adopted to resolve the dispute.

10. Expiration or termination of this Agreement does not relieve either party of any obligation which arises before expiration or termination, including without limitation obligations for payment and reporting. Any provision of this Agreement which contemplates performance or observance subsequent to any termination or expiration of this Agreement shall survive any termination or expiration of this Agreement and continue in full force and effect.

11. If any provision of this CDA is found to be unenforceable, such provision will be limited or deleted to the minimum extent necessary so that the remaining terms remain in full force and effect.

12. No waiver of any term, provision or condition of this CDA, whether by conduct or otherwise, in any one or more instances, shall be deemed to be or construed as a further or continuing waiver of the same term, provision or condition, or of any other term, provision or condition of this Agreement.

13. No Party shall be liable for any failure to perform as required by this CDA to the extent such failure to perform is due to circumstances reasonably beyond such Party's control, including, without limitation, labor disturbances or labor disputes of any kind, accident, civil disorders or commotions, acts of aggression or terrorism, acts of God, energy or other conservation measures imposed by law or regulation, explosions, failure of utilities, mechanical breakdowns, material shortages, disease, or other such occurrences.

SIGNATURE PAGE TO FOLLOW

IN WITNESS of this Agreement, the parties have executed this Agreement through their duly authorized representatives.

**Provider**	**Recipient**
By: __________________________________	By:__________________________________
Name: _______________________________	Name________________________________
Title:_________________________________	Title:_________________________________
Date: ________________________________	Date: ________________________________

The Recipient PI and Providing PI, by affixing their signatures below, acknowledge that they have read, understood, and agree to comply with the terms of this Agreement.

**Provider PI**	**Recipient PI**
By: __________________________________	By:__________________________________
Name: _______________________________	Name________________________________
Title:_________________________________	Title:_________________________________
Date: ________________________________	Date: ________________________________

PERCH CONSORTIUM
LIST OF PERCH DATASETS

The following is the complete list of PERCH datasets. Raw Datasets will not be shared except in unique circumstances for which no other reasonable acceptable solution is possible. Analytic Datasets appended with select additional variables required for an analysis will be prepared and provided to investigators. See **Annex 1** for additional details.

- Analytic and Results Datasets:
  - Lab
  - Clinical
  - *S. pneumoniae* serotyping
  - *H. influenzae* serotyping
  - PCR Testing Results
  - Raw and Confirmatory Lab Testing Results
- Raw Datasets:
  - CRF 01: Case Admission and Consent
  - CRF 01: Case Screening
  - CRF 01: Case Screening Clinical Exam
  - CRF 01A: Control Screening and Eligibility
  - CRF 01B: HIV+ Control Screening and Eligibility
  - CRF 03: Clinical History
  - CRF 03: Immunization History
  - CRF 04: Case Clinical Assessment
  - CRF 04A: Control Clinical Assessment
  - CRF 05: Demographics and Household
  - CRF 05: Environment and Healthcare
  - CRF 05: Household Income and Birth Milestones
  - CRF 06: Case Specimen Collection: Blood NP/OP Urine
  - CRF 06A: Control Specimen Collection: Blood NP/OP Urine
  - CRF 07: Case Specimen Collection: Induced Sputum
  - CRF 07ETT: Case - ETT Specimen
  - CRF 07GA: Case - Gastric Aspirate
  - CRF 08: Case CXR
  - CRF 08A: Chest X-Ray Evaluation
  - CRF 09: Case Specimen Collection: Lung Aspirate
  - CRF 10: Case - Pleural Fluid
  - CRF 11: Case Admission Medications
  - CRF 12: Case 24/48-Hour Follow-up
  - CRF 13: Case Discharge
  - CRF 14: Case 30-Day Follow-up
  - CRF 15: Case Specimen Collection: Consent
  - CRF 15: Case Specimen Collection: Post-Mortem Lung Biopsy

- CRF 16: Case Serious Adverse Event
- CRF 17: Case Mortality
- CRF 18: Study Completion/Termination
- CRF 19: Lab Reception
- CRF 19OTH: Lab Reception: Other Specimen
- CRF 19PM: Lab Reception: Post Mortem Specimen
- CRF 20: Lab Result: Blood Culture
- CRF 20: Lab Result: Blood Culture: Additional Tests
- CRF 21: Lab Result: Pneumococcal PCR
- CRF 22: Lab Result: Antibiotic Activity
- CRF 23: Lab Reception: Core Blood Tests - Cases
- CRF 23: Lab Reception: Core Blood Tests - Controls
- CRF 24: Lab Result: NP Culture
- CRF 25: Lab Result: Multiplex PCR
- CRF 26: Lab Result: Induced Sputum Micro-Culture
- CRF 26: Lab Result: Induced Sputum: Additional Tests
- CRF 27: Lab Results: TB Testing
- CRF 28: Lab Result: Pleural Fluid - Lung Aspirate
- CRF 28: Lab Result: Pleural Fluid: Additional Tests
- CRF 29: Lab Result: PCP Staining/Fluor Results
- CRF 30: Participant Event
- CRF 30A: Site Event
- CRF 31: Case Pre-Screening
- CRF 31Ai: EPI Control Screening
- CRF 31Aii: DSS Control Pre-Screening
- CRF 31B: HIV+ Control Screen
- CRF 32: Lab Result: Post-Mortem Lung Biopsy
- CRF 32: Lab Result: Post-Mortem Lung Biopsy: Additional Tests
- CRF 33: Histology Result: Post-Mortem Lung Biopsy

Note: The details in this appendix may be updated from time to time without formal amendment to the Agreement.

PERCH Dataset Access Request Form

To request access to the PERCH data, please complete applicable fields of **Section A** (contact information and Data Analysis Plan). Use the Data Analysis Plan (DAP) to outline the purpose of your request and analysis. Please submit the completed forms to Chrissy Prosperi (cprospe1@jhu.edu) and Melissa Higdon (mhigdon@jhu.edu). The Core team will contact you with any questions or concerns. If approved, you will be asked to complete **Section C** to identify the specific datasets or variables requested and sign a **Confidential Disclosure Agreement** prior to distribution of datasets. Attach this completed document as **Exhibit A** to the **Confidential Disclosure Agreement**. The Core team is available to assist in identifying datasets and variables to support the analysis.

Section A:

Contact details (* required)

Name*: Click here to enter text.

Title*: Click here to enter text.

PERCH Site Affiliation (N/A if non-PERCH Investigator)*: Click here to enter text.

Institution*: Click here to enter text.

Address: Click here to enter text.

Ph: Click here to enter text.

Email*: Click here to enter text.

PERCH Dataset Access Request Form

**Analysis #** (to be completed by PERCH Core)	
**Data Request Study Title Name (e.g., DAP Name)**	
**Analysis Team**	**Analysis Lead(s)**:<br>**Analysis collaborator(s)**:<br>**Coordinator** (if unknown, will be assigned following CORE review):<br>**Programmer** (indicate CORE/Emmes if support is required):
**Authorship Category Level** (to be completed by PERCH Core)	
**Objective(s)**	
**Informs (e.g., other analyses)**	
**Hypothesis or description of the problem**	
**Biologic rationale**	
**Case Definition** (Eligibility)	
**Data Sources** (clinical, lab, etiologic results, risk factors, etc.)	
**Specimen results included in analysis (or N/A)**	
**Analysis Plan**	
**Analytic Output**	
**Key Supporting Analyses (or N/A)**	
**Sub-analyses and Stratifying Variables**	
**Potential that data will be used for**	

**Commercial Purposes? Note: requires separate written agreement.**	

Example Key Tables and Figures (can also be sketched out by hand; not required for initial submission)

Section B (FOR INTERNAL PERCH USE ONLY)

**PERCH Data Access Committee decision:**	☐ Approved<br>☐ Approved, pending changes<br>☐ Denied
**If “Approved, pending changes” or “Denied”, describe changes or reason for denial:**	

PERCH Dataset Access Request Form

Section C:

Please indicate which PERCH Datasets you are requesting:

☐ Clinical analytic dataset

☐ Laboratory analytic dataset

☐ Pneumococcal serotyping analytic dataset

☐ *Haemophilus influenzae* serotyping analytic dataset

☐ Other (indicate any additional fields/forms required): Click here to enter text.

Please specify any additional information regarding this data request:

☐ All sites

☐ Select sites: Click here to enter text.

☐ Specific subpopulation (e.g., age group, enrollment period, cases only): Click here to enter text.

☐ Data format (e.g., SAS, Stata, SPSS): Click here to enter text.

☐ Other:

Note: The details in this appendix may be updated from time to time without formal amendment to the Agreement.

PERCH Ancillary Study or Collaboration Request Application
Request for Specimens

PERCH aims to conduct a high-quality, rigorous study of the etiology of severe and very severe pneumonia among children in developing countries. While the focus of PERCH is to meet the primary objectives laid out in the project protocol and proposal, the PERCH Consortium also recognizes that the 'platform' of the study provides some unique and/or highly efficient opportunities to investigate related or ancillary questions to the primary objectives. These 'ancillary studies' may be either collaborations within the PERCH Consortium or in collaboration with external collaborating institutions.

This form is designed to serve as a way for PERCH to assess and track specimen requests for ancillary studies and collaborations. All requests for specimens should have one of these forms completed and approved by the PERCH Biorepository Specimen and Access Committee before any work or commitments for work are undertaken. There is a **2 page limit** for the form. Please complete Section A and submit completed forms, with the investigator's *curriculum vitae*, to Chrissy Prosperi (cprospe1@jhu.edu) and Melissa Higdon (mhigdon@jhu.edu).
If approved, complete Section C to document the shipment terms and the handling of specimens upon termination agreed upon by the Provider and Recipient. A **Material Transfer Agreement** must be signed prior to the shipment of specimens. Attach this completed document as **Exhibit A** to the Material Transfer Agreement.

PERCH Ancillary Study or Collaboration Request Application
Request for Specimens

Section A

**Name of person submitting the form**	
**Specimen Request Study Title Name**	
**Date**	
**Phone**	
**Email**	
**Proposed lead institution**	
**PERCH Consortium Member request?**	
**Proposed start date and end date for proposed study**	
**Is there an important deadline related to this proposal?**	
**<u>Type of Proposed Collaboration</u>**<br>(single site or multiple site; if multiple, indicate site names)	
**Name and Affiliated Institution(s) of other Collaborators**	
**<u>Funding</u>**<br>**Do you anticipate the collaboration will need new funds?**<br><br>**If yes, are you requesting funds from the PERCH Project?**<br>If yes, please submit a proposed budget.	
**<u>IRB Approval</u>**<br>**Do you anticipate separate IRB approval for the collaboration?**	
**<u>Benefit to PERCH</u>**<br>(List what PERCH may expect from this collaboration; particularly trainings, internships, or publication opportunities. When these might be available for young investigators and those at PERCH sites please make this explicit)	
**<u>Type of request of PERCH (indicate all that apply)</u>**<br>**A) Biological materials**<br>**B) Data (clinical/lab)**<br>**C) Other request of PERCH**	
**<u>Request For Materials:</u>**<br>Describe the types, numbers and volume of biological materials that would be needed. Indicate if requesting specimens from cases/controls (or both), a specified time period or other special request.	
**<u>Request for Data:</u>**<br>Provide a brief description of the types of clinical, lab or other data requested. Note, a separate Dataset Access Request Form and Confidential Disclosure Agreement must be completed prior to distribution of data.	
**Clinical Data Requested:**	
**Lab Data Requested:**	
**Other data request:**	

Request for Specimens

**Name of Institution/Company responsible for paying specimen/distribution fees**	
**Name and address of Institution/Company to which biological materials would be shipped**	
**Purpose/Hypothesis**	
**Will the Materials be used now or in the future for Commercial Purposes?** If yes, separate written agreement will be required.	
**Briefly list primary research aims**	
**Briefly outline methods (epi, stats, lab, or others)**	
**Other supporting materials**	

Section B (FOR INTERNAL PERCH USE ONLY)

**PERCH Biorepository Specimen and Access Committee decision:**	☐ Approved<br>☐ Approved, pending changes<br>☐ Denied
**If "Approved, pending changes" or "Denied", description of changes or reason for denial:**	

Section C

**Describe the terms for shipment of specimen, as**	

**agreed upon by Provider and Recipient**	
**Handling of specimens following termination of Agreement, as agreed upon by Provider and Recipient:**	☐ Destroy materials<br>☐ Return materials to Recipient

Note: The details in this appendix may be updated from time to time without formal amendment to the Agreement.
