# Supplementary material for: Pneumococcal colonization prevalence and density among Thai children with severe pneumonia and community controls
Source: PLoS One. 2020 Apr 29;15(4):e0232151. doi: 10.1371/journal.pone.0232151 (PMC7190126; doi:10.1371/journal.pone.0232151)
Supplement: S1 File — (PDF) [file pone.0232151.s005.pdf]

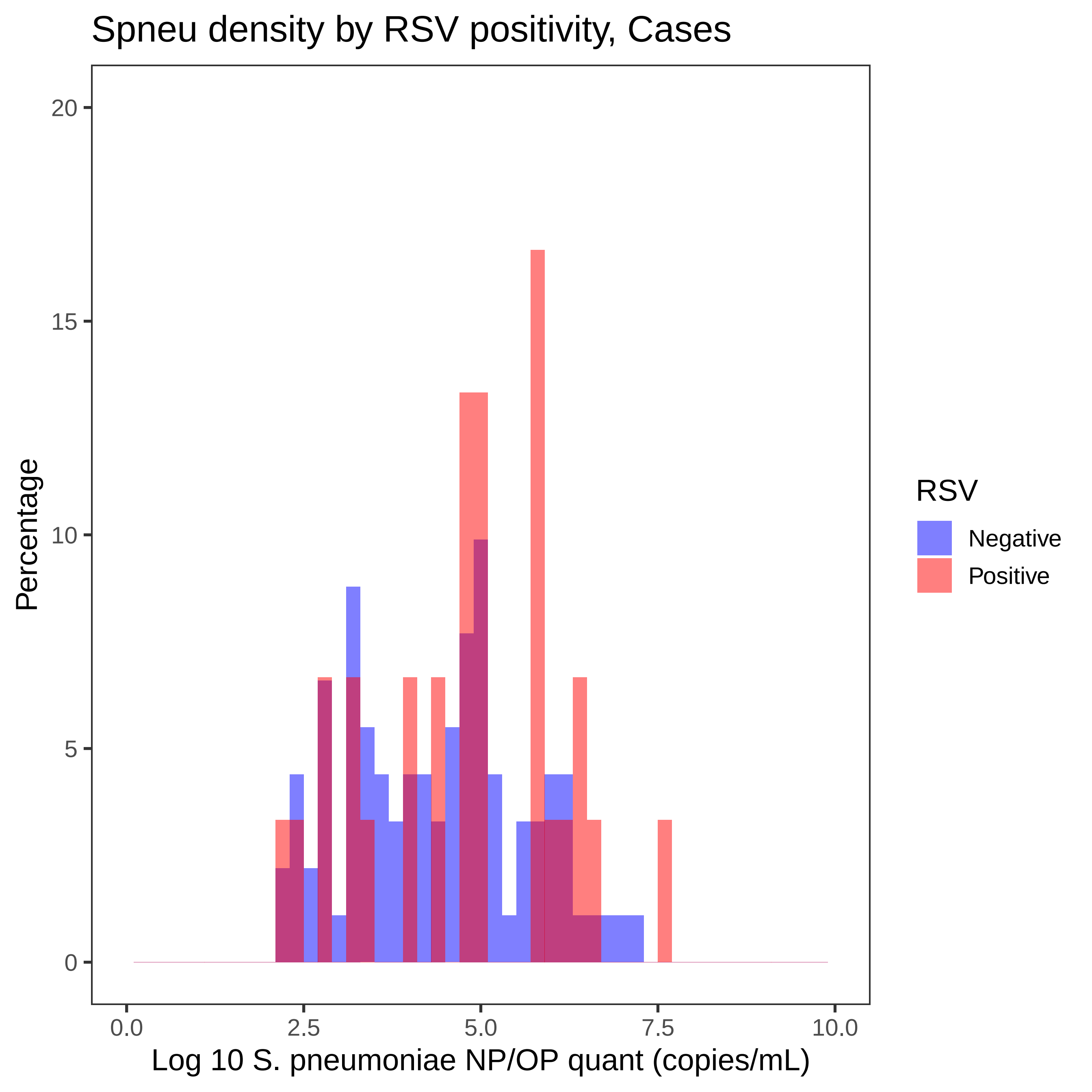

Spneu density by RSV positivity, Cases
Percentage
0
5
10
15
20
0.0
2.5
5.0
7.5
10.0
Log 10 S. pneumoniae NP/OP quant (copies/mL)
RSV
Negative
Positive

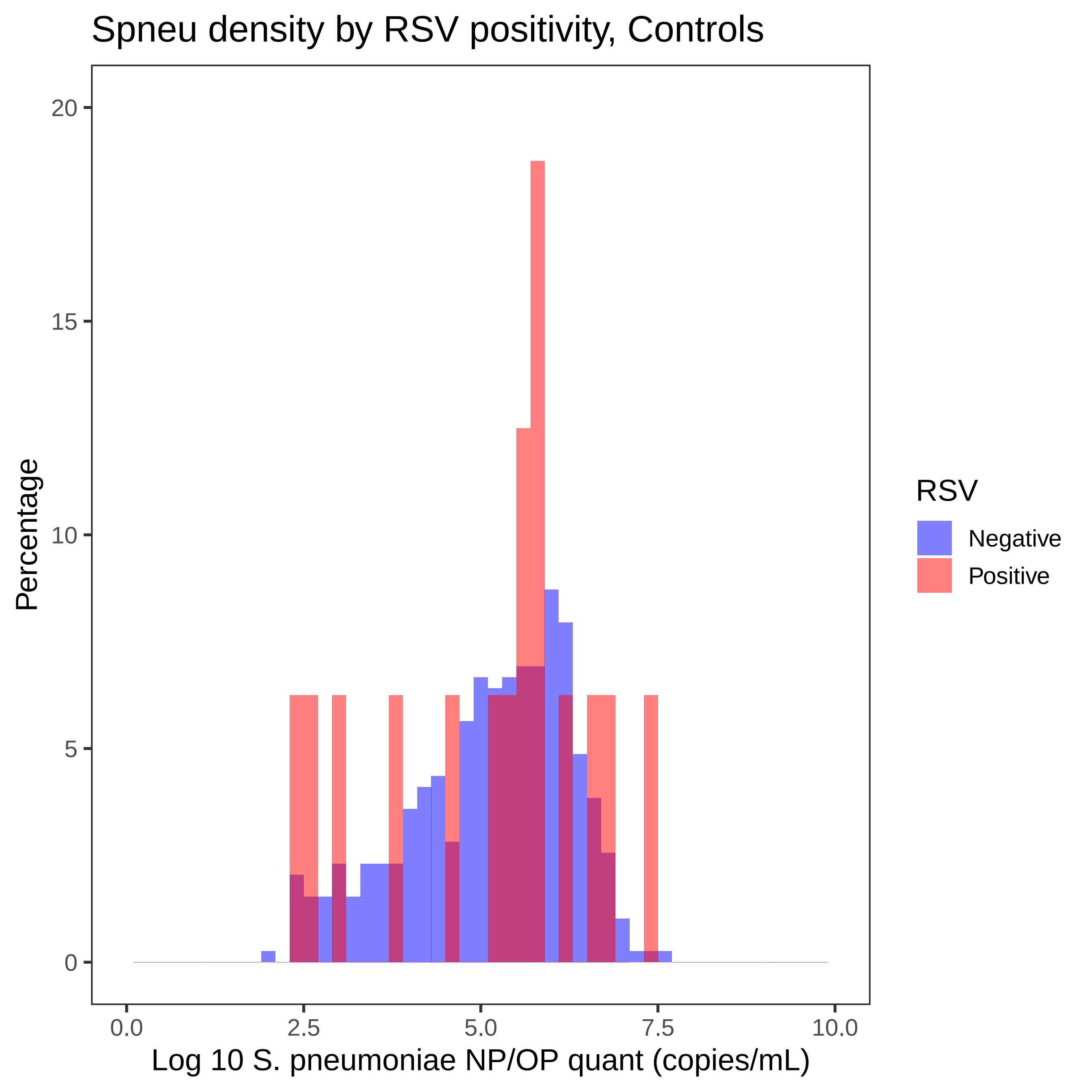

Spneu density by RSV positivity, Controls
Percentage
20
15
10
5
0
0.0
2.5
5.0
7.5
10.0
Log 10 S. pneumoniae NP/OP quant (copies/mL)
RSV
Negative
Positive

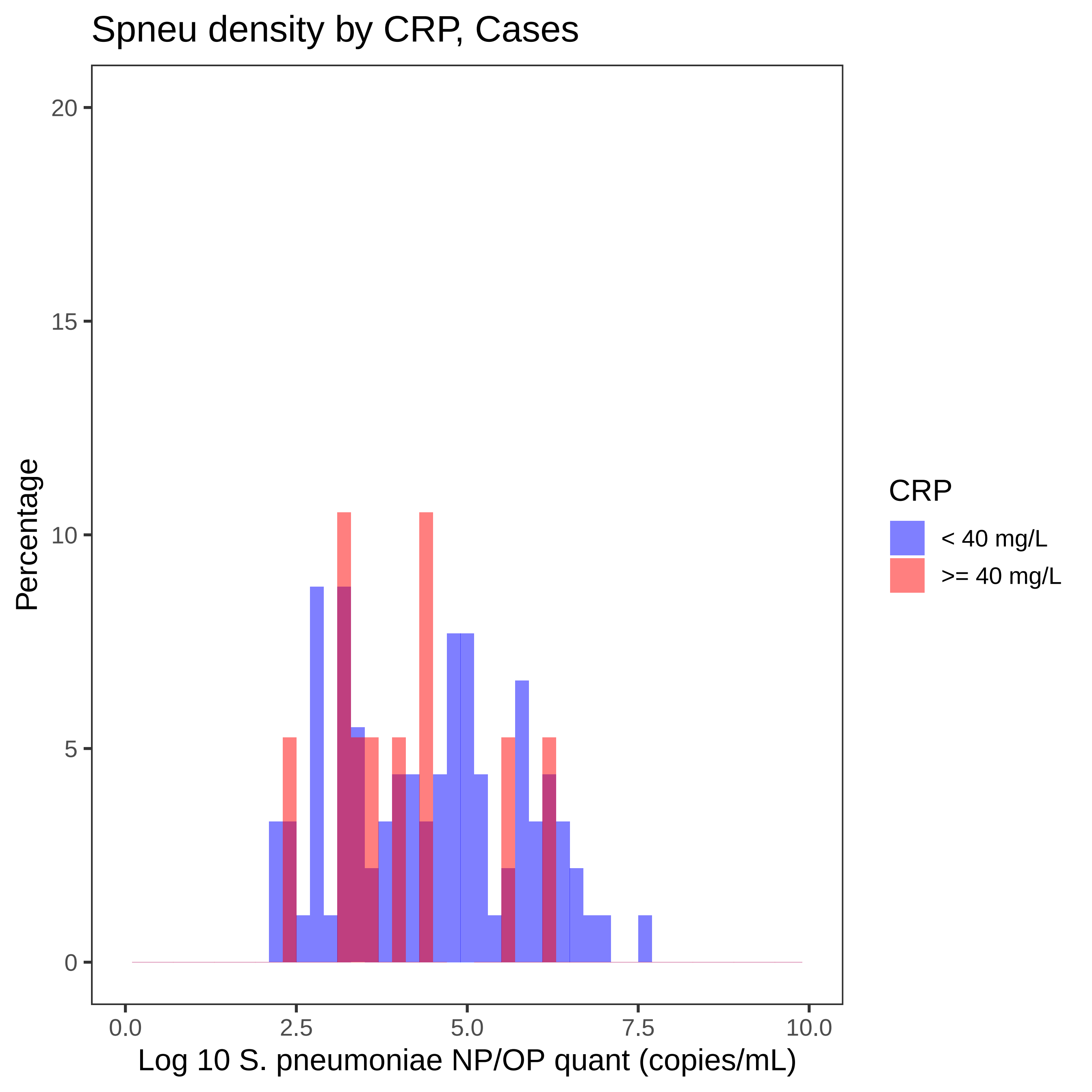
Spneu density by CRP, Cases
Percentage
0
5
10
15
20
0.0
2.5
5.0
7.5
10.0
Log 10 S. pneumoniae NP/OP quant (copies/mL)
CRP
< 40 mg/L
>= 40 mg/L

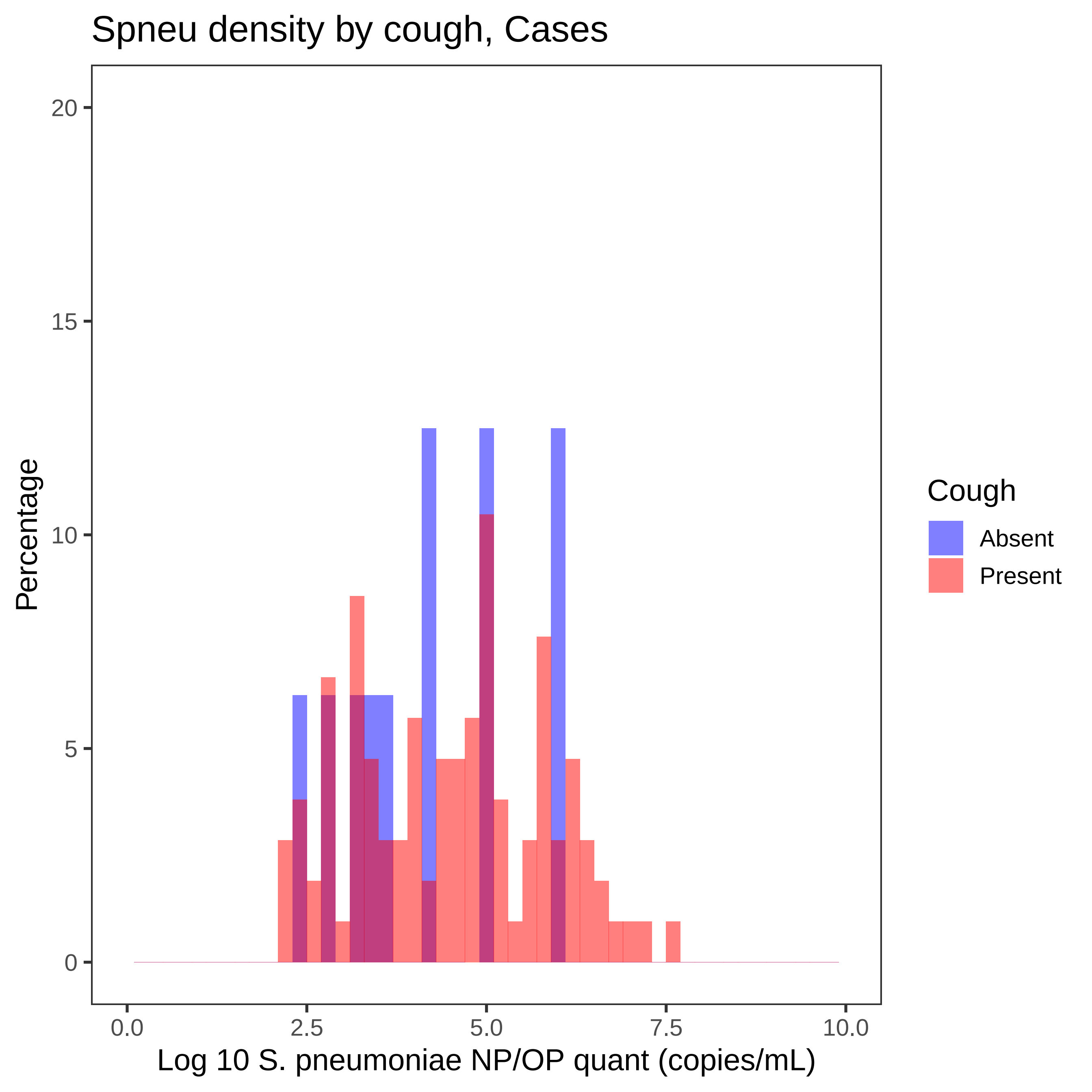
Spneu density by cough, Cases
Percentage
20
15
10
5
0
0.0
2.5
5.0
7.5
10.0
Log 10 S. pneumoniae NP/OP quant (copies/mL)
Cough
Absent
Present

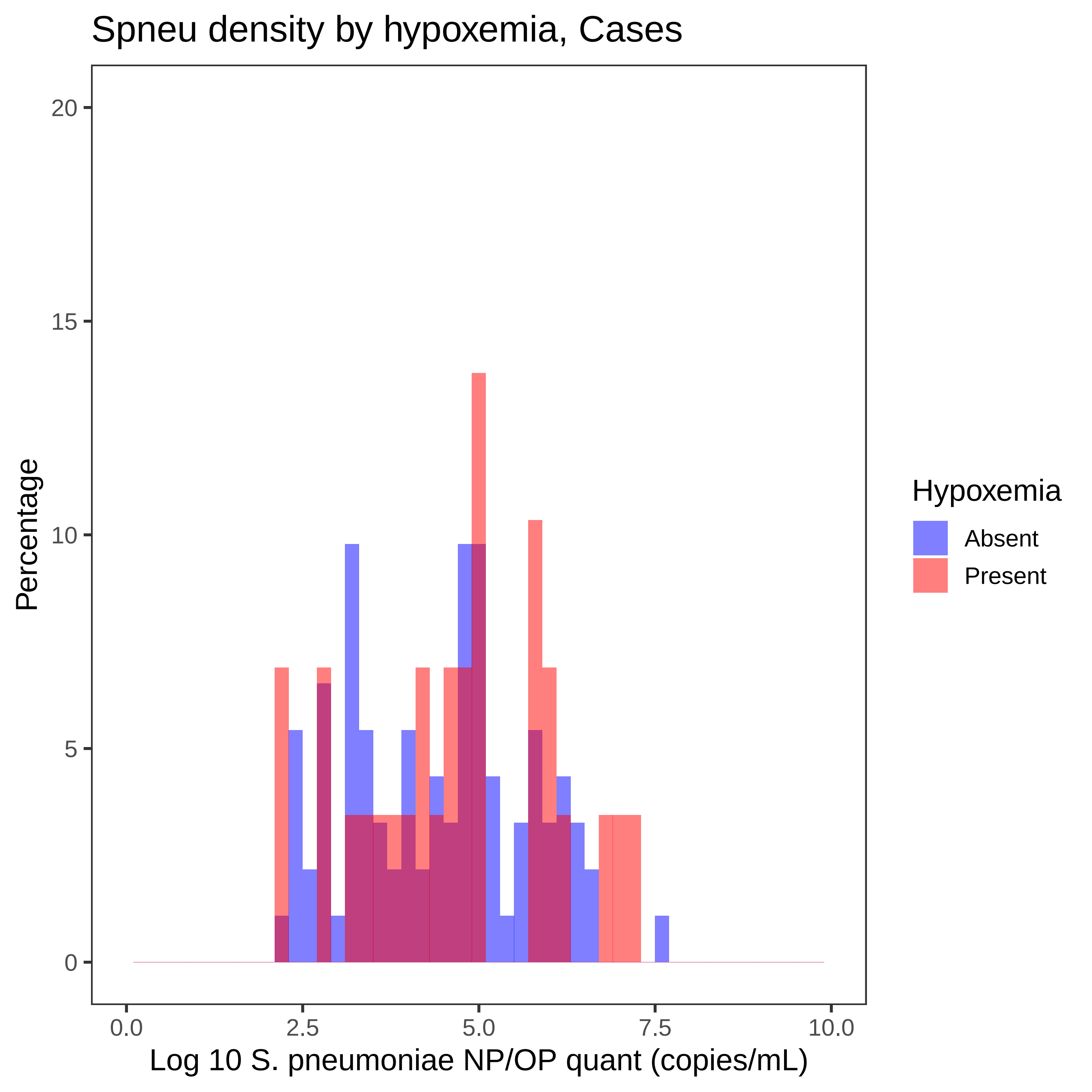
Spneu density by hypoxemia, Cases
Percentage
20
15
10
5
0
0.0
2.5
5.0
7.5
10.0
Log 10 S. pneumoniae NP/OP quant (copies/mL)
Hypoxemia
Absent
Present

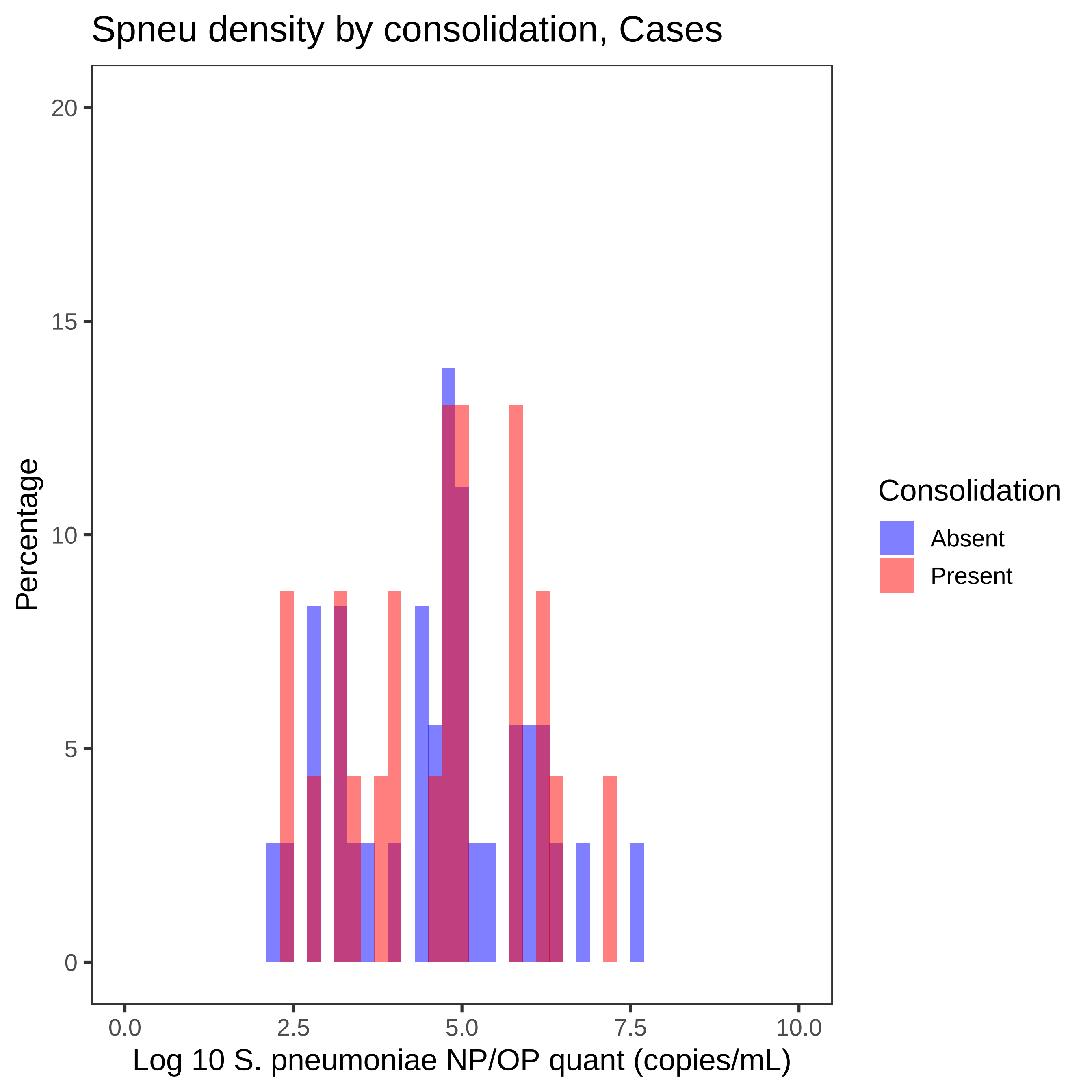

Spneu density by consolidation, Cases
Percentage
0
5
10
15
20
0.0
2.5
5.0
7.5
10.0
Log 10 S. pneumoniae NP/OP quant (copies/mL)
Consolidation
Absent
Present

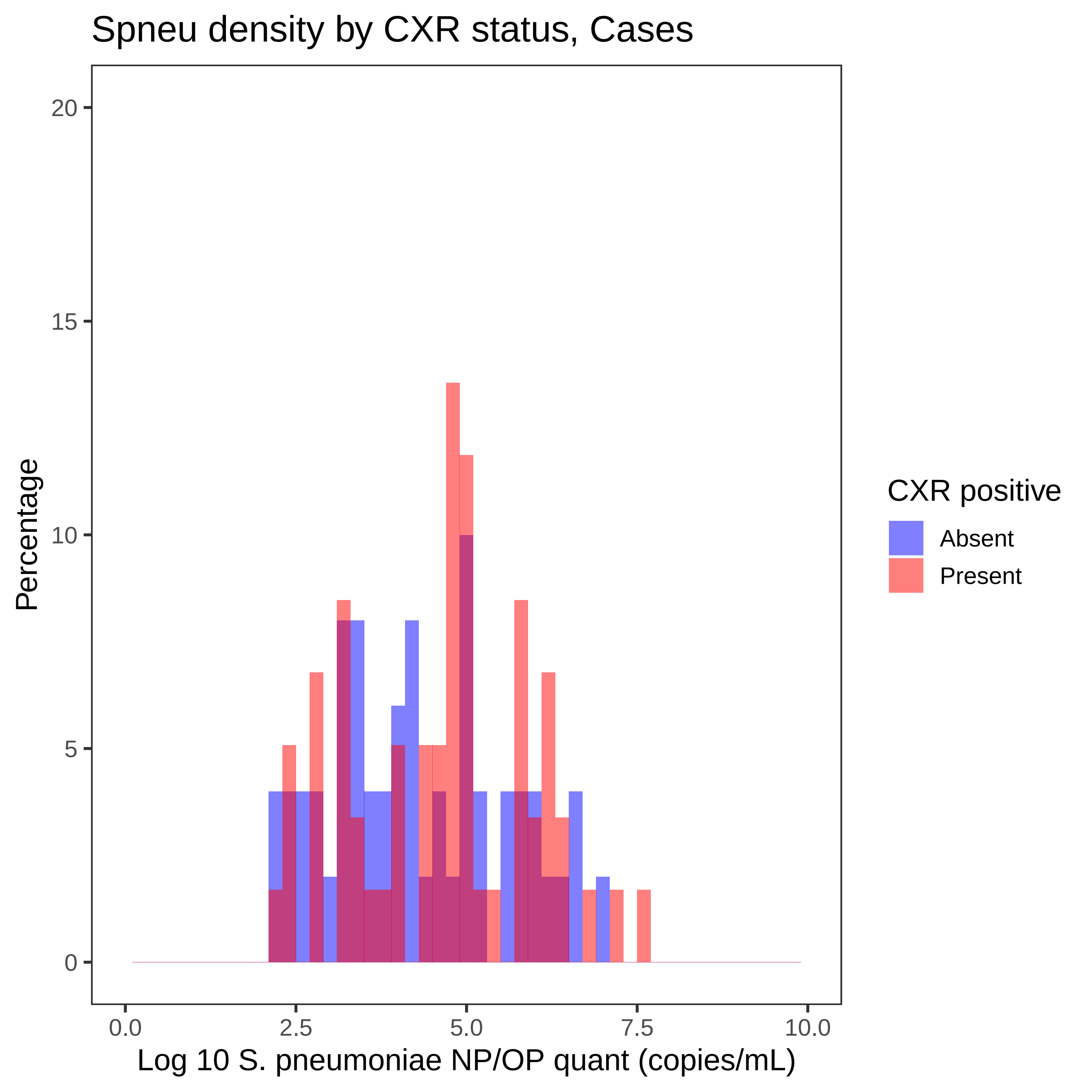

Spneu density by CXR status, Cases
Percentage
20
15
10
5
0
0.0
2.5
5.0
7.5
10.0
Log 10 S. pneumoniae NP/OP quant (copies/mL)
CXR positive
Absent
Present

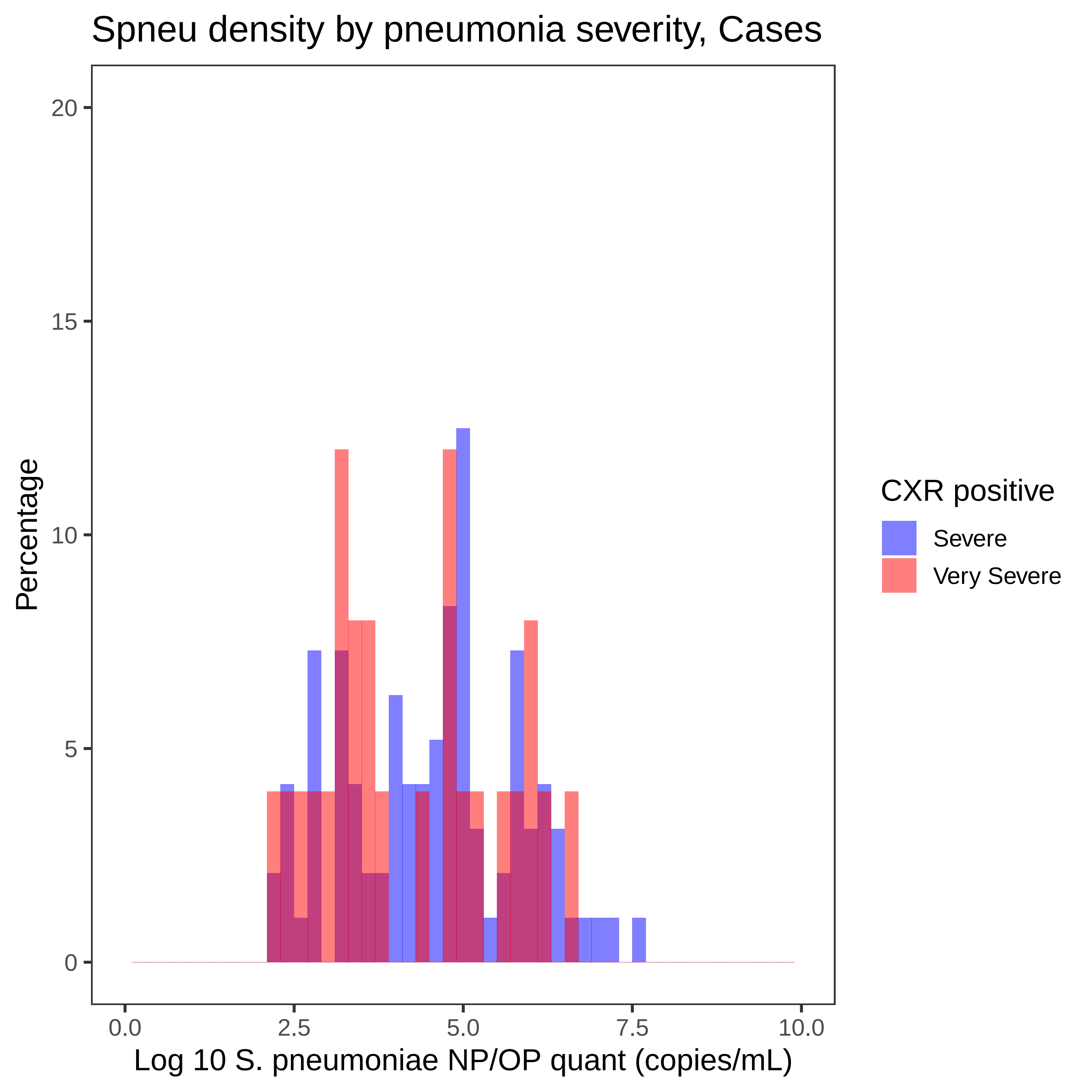

Spneu density by pneumonia severity, Cases
Percentage
Log 10 S. pneumoniae NP/OP quant (copies/mL)
0
5
10
15
20
0.0
2.5
5.0
7.5
10.0
CXR positive
Severe
Very Severe

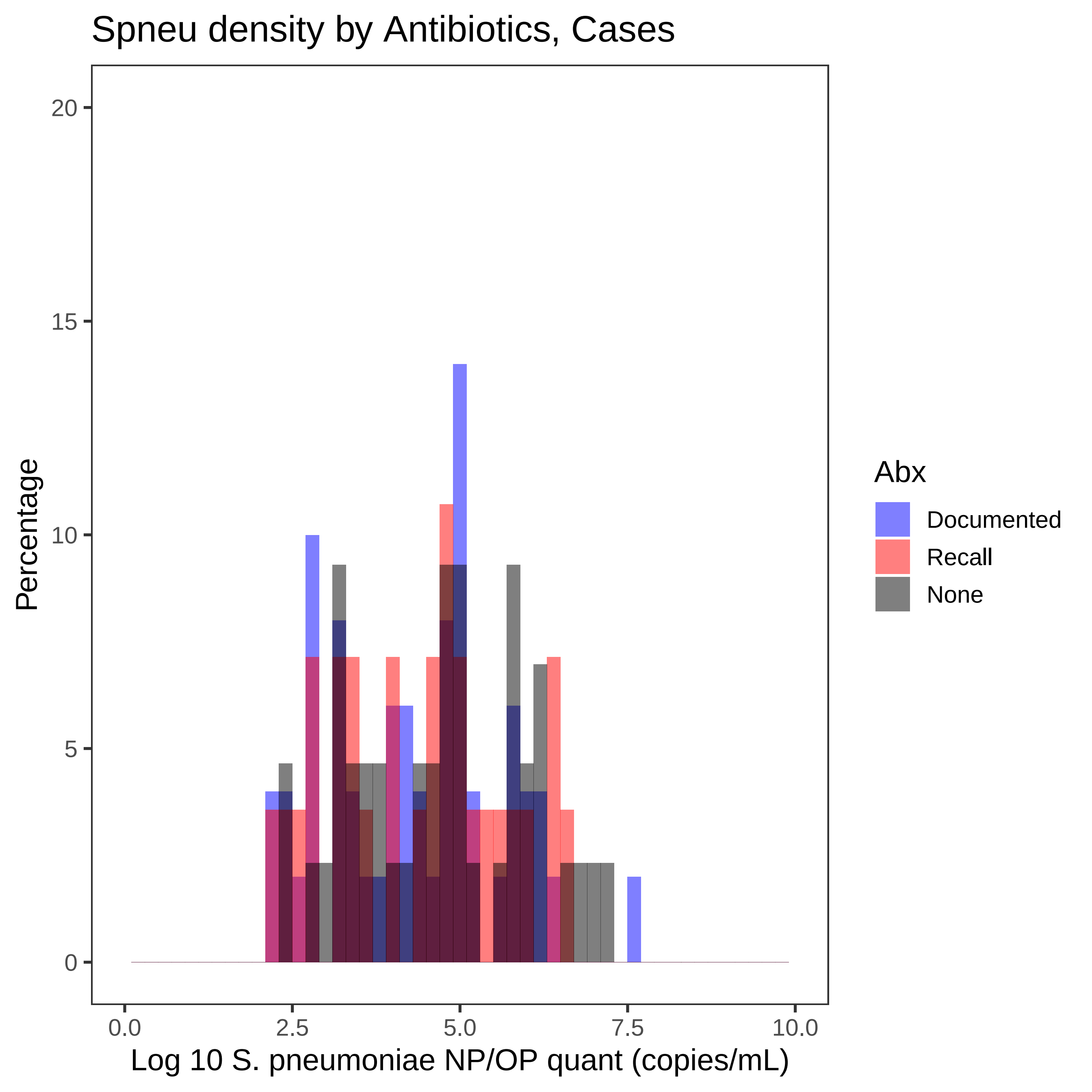
Spneu density by Antibiotics, Cases
Percentage
0
5
10
15
20
0.0
2.5
5.0
7.5
10.0
Log 10 S. pneumoniae NP/OP quant (copies/mL)
Abx
Documented
Recall
None
