# Supplementary material for: Pneumococcal colonization prevalence and density among Thai children with severe pneumonia and community controls
Source: PLoS One. 2020 Apr 29;15(4):e0232151. doi: 10.1371/journal.pone.0232151 (PMC7190126; doi:10.1371/journal.pone.0232151)
Supplement: Attachment — Submitted filename: review_piralam.doc [file pone.0232151.s006.doc]

Piralam and coll. assessed pneumococcal nasopharyngeal (NP) colonization in hospitalized children with pneumonia and in controls in Thailand. They also assessed pneumococcal colonization density among young children hospitalized with pneumonia and the association with characteristics of pneumonia, and distribution of serotypes. They used the data of the large multicentre PERCH study.

224 pneumonia cases and 659 community controls were included in the 2 study sites; both located in Thailand. The results showed a lower proportion of NP colonization and a lower median colonization density in cases compared to controls. No statistical differences were found in sub-groups analyses for colonization density.

Nowadays, PCR on NP samples cannot be used as a diagnostic tool as it has an uncertain value for individual diagnosis. Association between pneumococcal NP colonization density and pneumonia is, to my opinion, a topic of great interest. As a lot of asymptomatic controls have a NP colonization; a high amount of *S. pneumoniae* might be associated with pneumonia (as the authors mentioned in Introduction section l.93 réf 7-11).

The presented results are unexpected and hypotheses to explain them should be clearly formulated. Overall analyses of the PERCH study showed an association between pneumococcal colonisation density and microbiologically-confirmed pneumonia and the results in the Thai sub-group are opposite (réf 7). Then, potential bias should be carefully raised. I think that the manuscript can easily be improved (especially Results and Discussion sections). Be careful with typographical errors.

**Major comments**

1. Table 2 has to be improved, particularly to help the reader to understand quickly the Table. It is unclear. For example, in each line of the study characteristics, there are several p-value (for example: age P=0.012, P=0.31 etc.). It is not clear at all what mean these p-values and what is compared in this Table. The authors may add a new column for p-value and precise in header information what is compared. The authors may also perhaps split the Table in 2 Tables as some characteristics were not assessed in Controls. I particularly don’t understand to what refer p-values for characteristics not measured in controls (such as hypoxemia).
2. The authors may add some characteristics in Table 1 (or appendix …) in order to better describe the studied population (ex: for cases % detection of pneumococcus in blood by PCR, symptoms, etc).
3. The text of the results section is quite difficult to read with numerous imprecisions (see minor comments). You may try to clarify and focus on the interesting results.
4. Figure 1 is not understandable. Some serotypes are missing on the x-axis. The authors do not mention if some proportions are different between cases and controls. What mean NT?
5. How was collected the variable “prior antibiotic exposure”? If the quite low proportion of NP carriage of pneumococcus in cases might be explained by a higher proportion of antibiotic intake in cases, then the proportion of NP carriage should be higher in the group without previous antibiotic intake compared to the group without antibiotic intake. What hypotheses can be formulated to explain this?
6. Discussion p. 22 l.374 and after. Did you find other studies showing a higher pneumococcal density in healthy controls compared to pneumonia cases? I think that you have to carefully mention potential alternative explanations to explain this unexpected result: were operators (clinicians who collect the swab on subjects) the same between the hospital study site and community? If controls may have RTI, is it possible that the sensitivity of the swab was higher/increased in controls compared to the sensitivity in pneumonia cases (in the case of a higher amount of mucus, etc)?
7. I think that you have to clearly mention the proportion of cases and controls vaccinated with PCV. If no cases and controls were vaccinated because PCV was not in the national immunization program during the stud, you should clearly mention it.
8. The authors have to clearly present the potential bias. What about a selection bias in the study if the controls are all included in a same population with a high prevalence of NP carriage of *S. pneumoniae*?

**Minor comments**

1. P.3 l.67 10^3^ and not 103
2. The authors precise that they performed binary logistic regression adjusted for age; as the study was frequency matched for age. You mean that the study was not paired matched, and then, that it was not possible to perform a conditional logistic regression?
3. P. 17, l.261 I think that it is better to mention IQR with a “-“ and not “,” (for example 2.1-222.3)
4. P.17, l.262 is it possible to add the p-value between proportion of cases and control above the threshold identified in the overall PERCH analysis?
5. P.17, l. 264 mL instead of ml
6. P.17, l. 266 I don’t understand to what the p-value (p=0.31) refers? It may be better to mention “The density is highest […] even if not significant” or mention the p-values after the sentence “none of these differences reached statistical significance”
7. P.17, l. 274 Only 3 cases had pneumococcus detected by PCR in whole blood? You may mention in the population description
8. P. 17, l. 275 Do you mean “were colonized in NP” ?
9. P. 17, l. 278 Do you mean “in whole blood by PCR”?
10. P. 18, l. 286 “with NP colonization”
11. Figures 1 & 2 the caption of y-axis “percent of children” is not clear. You might mention “percent of children with pneumococcal NP colonization”
12. P. 18, l. 299 I think that it is better to mention 95% CI with a “-“ and not “,”
13. P. 19, l. 301 “7 of the 8 most frequent serotypes” instead of “the top 8 serotypes”
14. P. 20, l. 339 remove “%”
15. P. 24, l. 424 “though …. unresolved serotypes”. This sentence is difficult to understand, you may rewrite it. I don’t know the meaning of the word “apporportioned”. Do you mean “apportioned”? What are the “unresolved serotypes”?
