# Supplementary material for: Pneumococcal colonization prevalence and density among Thai children with severe pneumonia and community controls
Source: PLoS One. 2020 Apr 29;15(4):e0232151. doi: 10.1371/journal.pone.0232151 (PMC7190126; doi:10.1371/journal.pone.0232151)
Supplement: Attachment — Submitted filename: Response to Reviewers_20200214.docx [file pone.0232151.s008.docx]

14 February 2020

RE: Manuscript revision: Pneumococcal colonization prevalence and density among Thai children with severe pneumonia and community controls (PONE-D-19-29663)

Dear Editor:

Thank you for your quick review. We are submitting a revised version of our manuscript entitled: “Pneumococcal colonization prevalence and density among Thai children with severe pneumonia and community controls” (PONE-D-19-29663) for consideration for publication in the PLOS ONE journal.

I would like to provide a point-by-point response to the two remaining requests related to our manuscript:

1. Major comment no. 2; the authors may add some characteristics in Table 1 (or appendix …) in order to better describe the studied population (ex: for cases % detection of pneumococcus in blood by PCR, symptoms, etc).

**Response:** We have added key laboratory and clinical characteristics to table 1, which complements the information in table 2 (stratified by pneumococcal NP/OP positivity). These variables include sub-site, sex, pneumococcal positivity in the NP/OP by PCR and culture, pneumococcal positivity in whole blood, and clinical symptoms (cough, fever). Also, a full description of all cases and controls in Thailand is pending publication. If that paper will be accepted before publication of this paper, we will add a reference to that paper into our paper under review at PLoS One.

2. Major comment no. 8; the authors have to clearly present the potential bias. What about a selection bias in the study if the controls are all included in a same population with a high prevalence of NP carriage of *S. pneumoniae*?

**Response:** We recognize that all case-control studies are subject to potential selection bias. To acknowledge this point, we added the following sentences to the limitations paragraph in the Discussion section (lines 412-419): Third, all case-control studies are subject to potential selection bias related to control selection. We believe that selection bias was minimized by our approach to control selection; community controls were randomly selected from comprehensive lists of children aged 1 - 59 months drawn from health services registries, which include virtually all children in each province, and were enrolled year round and frequency matched to the age-group distribution of the cases on a monthly basis. Therefore, our controls represented an unbiased community comparison group in terms of pneumococcal colonization prevalence.

Regarding to the suggestion to deposit our laboratory protocols in [protocols.io](http://protocols.io), please note that all study protocols and standard operating procedures, including laboratory SOPs, were published and available online at <https://www.jhsph.edu/ivac/resources/perch-background-and-methods/> which cited as reference number 26 in the line 139.

We hope that the revised manuscript addresses all comments and meets the high standards of your journal. Thank you for your consideration.

Sincerely yours,

Barameht Piralam

Address: Nakhon Phanom Provincial Health Office

356 Abhibanbancha Rd, Naimuang subdistrict, Muang district,

Nakhon Phanom province, Thailand

Email: [baramehtp@ieipnp.go.th](mailto:baramehtp@ieipnp.go.th), baramehtp@gmail.com

Phone: [+6692-](tel:%2B6692-991-4915)991-4915
